# *N*‐Sulfonyl Azalevoglucosan: A Versatile Platform for the Synthesis of Polysubstituted Piperidines

**DOI:** 10.1002/chem.71491

**Published:** 2026-07-28

**Authors:** Dylan Yorga, Jérôme Marrot, Ana Poveda, Nicolas Auberger, Jesús Jiménez‐Barbero, Jérôme Désiré, Yves Blériot

**Affiliations:** ^1^ Groupe Glycochimie, Equipe OrgaSynth, IC2MP‐UMR CNRS 7285 Université de Poitiers Poitiers cedex France; ^2^ Institut Lavoisier de Versailles, UVSQ, CNRS, UMR 8180 Université Paris‐Saclay Versailles cedex France; ^3^ ClC bioGUNE Basque Research and Technology Alliance (BRTA) Derio Spain; ^4^ Ikerbasque Basque Foundation for Science Bilbao Spain; ^5^ Department of Organic & Inorganic Chemistry, Faculty of Science and Technology University of the Basque Country EHU‐UPV Leioa Spain; ^6^ Centro de Investigacion Biomédica En Red De Enfermedades Respiratorias Madrid Spain

**Keywords:** hemiaminal, iminosugar, levoglucosan, *N*‐sulfonyl, piperidine

## Abstract

Substituted piperidines are key synthetic fragments for designing drugs and play a significant role in the pharmaceutical industry. The reported synthetic methods allow access to mono‐, di‐, and tri‐substituted piperidines, but tetra‐ and penta‐substituted piperidines are scarce in the literature. In this study, exploiting the inherent chirality of iminosugars, we report the synthesis of a levoglucosan mimic in which the endocyclic oxygen of the pyranose ring has been replaced by a *N*‐sulfonyl group. The trans diaxial arrangement of its hydroxyl groups allows navigation on the piperidine ring to iteratively install functional groups at C1, C2, C3, C4, and C6 positions using simple experimental protocols with minimal protecting group manipulation. The broad functional group scope combined with the high regio‐ and stereo‐selectivity make this approach an appealing process for diversity‐oriented synthesis of polysubstituted piperidines, including modified iminosugars. We expect this platform will support drug discovery efforts to accelerate the discovery of therapeutically relevant molecules.

## Graphical abstract

**Gain by strain**: The synthesis of a levoglucosan analog, in which the pyranose oxygen has been replaced by a nitrogen, allows navigation on the piperidine ring to iteratively install functional groups at C1, C2, C3, C4, and C6 positions using simple experimental protocols with minimal protecting group manipulation. The broad functional group scope combined with the high regio‐ and stereo‐selectivity pave the way to polysubstituted piperidines bearing five contiguous functions.

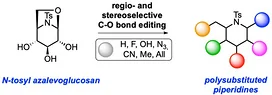

## 1 Introduction

The piperidine ring ranks amongst one of the most popular heterocycle motifs in FDA‐approved pharmaceuticals (Figure 1A) [1, 2]. Complex piperidines are becoming more popular [3] as drug discovery gradually moves toward nonplanar, more topologically complex cyclic molecular frameworks [4]. The modular synthesis of such scaffolds with precise stereochemical control is complex [5] and development of new synthetic methods for complex piperidines has been therefore continuously and actively pursued [6, 7, 8, 9, 10, 11]. The reported general methods include intramolecular cyclization, [12] intermolecular cycloaddition, [13, 14] dearomative saturation of pyridines [15, 16, 17], and direct C–H functionalization of simple piperidines, [18, 19, 20, 21] allowing access to mono‐, di‐, and tri‐substituted piperidines. However, reports of therapeutically relevant tetra‐ and penta‐substituted piperidines are scarce in the literature [22] (Figure 1B).

**SCHEME 1:**
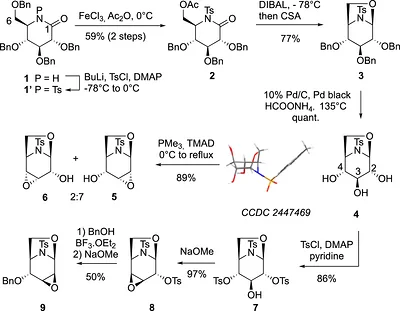
Synthesis of *N*‐tosyl azalevoglucosan **4** and reactive congeners **5–9**.

In the 60's, the group of Cerny has made a major contribution to the chemistry of carbohydrates, achieving the differentiation among secondary hydroxyl groups of comparable reactivity using levoglucosan, a conformationally locked 1,6‐anhydrosugar (Figure 1C) [23]. The 1,6‐anhydro core prevents protection of both 6‐OH and anomeric positions and facilitates navigation on the pyran ring to install functional groups using simple experimental protocols. Furthermore, the trans diaxial arrangement of the secondary hydroxyl groups allows a minimal use of protection/deprotection cycles and excellent regio‐ and stereo‐control. Since, levoglucosan and related Cerny epoxide [24] have been used by many groups to access carbohydrate derivatives of biological interest, including glycosaminoglycans, [25] iminosugars, [26] cyclodextrin analogs, [27] fluorinated monosaccharides [28], and glycosyl donors [29]. We postulate that exploitation of an “azalevoglucosan”, in which the pyran ring is replaced by a piperidine ring and the acetal bridge replaced by a hemiaminal, should enable the site‐selective C─O bond editing of the piperidine C2, C3, and C4 positions. In addition, ring opening of the hemiaminal bridge should allow the C─O bond editing of C1 and C6 positions, providing pentadifferentiated piperidines (Figure 1D).

**FIGURE 1:**
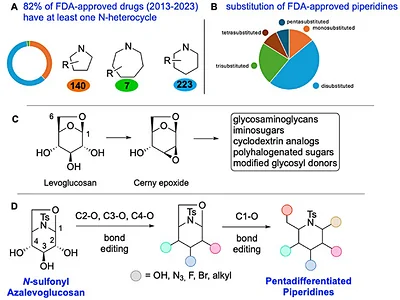
(A) Common nitrogen‐heterocycle motifs in FDA‐approved small‐molecule drugs. (B) Substitution pattern in US FDA‐approved pharmaceuticals containing piperidines. (C) Synthetic utility of levoglucosan and Cerny epoxide. (D) Overview of the current study.

## 2 Results and Discussion

Ideally, the synthesis of azalevoglucosan should start from naturally occurring nojirimycin, a glucopyranose analog [30]. However, its cost as bisulfite adduct ($1000/25 mgs) forced us to design an alternative route from tetra‐*O*‐benzyl‐d‐gluconolactam **1**, available in three steps and 64% yield from commercial tetra‐*O*‐benzyl glucose (Scheme 1) [31]. A robust *N*‐tosyl group, that should advantageously avoid formation of rotamers and lead to crystalline compounds, was installed to produce lactam **1’**. Its regioselective debenzylation at C6 position was achieved with FeCl_3_ in acetic anhydride [32] to furnish ester **2** (59% yield over two steps).

Bridging the C1 and C6 positions was performed via reduction of the lactam and ester with DIBAL at −78°C and subsequent treatment of the crude product with camphor sulfonic acid, to furnish bicyclic hemiaminal **3** (77% yield over two steps), via interception of the iminium intermediate by the primary alcohol [33]. Subsequent optimized hydrogenolysis combining Pd black, 10% Pd/C, and HCOONH_4_ quantitatively afforded *N*‐tosyl azalevoglucosan **4**. The structure of this bench‐stable bicycle was firmly established by x‐ray crystallography (CCDC 2447469), confirming the trans diaxial arrangement of the three secondary hydroxyl groups. Such derivative is unprecedented in the literature albeit some stereoisomers have been reported [34, 35, 36, 37, 38, 39, 40]. As for levoglucosan, a gradual reactivity of the three secondary OH groups in **4** was expected to access a range of Cerny‐like epoxides that are required to regio‐ and stereo‐selectively functionalize the piperidine ring. Satisfyingly, applying Mitsunobu conditions (PMe_3_, TMAD) [41] provided a mixture of 2,3‐ and 3,4‐exo‐epoxides **5** and **6** (CCDC 2487464) in 89% yield (7:2 ratio). In parallel, tosylation (TsCl, DMAP, pyridine) of triol **4** furnished the 2,4‐ditosyl derivative **7** in 86% yield, which afforded the 3,4‐endo‐epoxide **8** in 97% yield upon treatment with sodium methoxide. In addition, the 2,3‐endo‐epoxide **9** was obtained in 50% yield from epoxide **8** through opening with benzyl alcohol in the presence of BF_3_.OEt_2_ followed by treatment with NaOMe. With the four epoxides **5**, **6**, **8** and **9** and the ditosyl derivative **7** in hand, introduction of new functions through fluorination, azidation, and alkylation, either via alcohol SN_2_ substitution or regio‐ and stereo‐selective nucleophilic epoxide opening, was scrutinized. Indeed, piperidines bearing fluorine atoms have proved useful in pharmaceutical and agrochemical research [42]. Azide group has been extensively used as a masked amine and as partner for click chemistry reactions [43]. Alkylated piperidine motifs are often found in natural products [44].

Diversification of the C2 position [45] was first examined. Treatment of epoxyalcohol **6** with Tf_2_O followed by sodium azide gave the corresponding C2‐azide **10a** with retention of configuration (32% yield) along with a rearranged product **10b** (32% yield, CCDC 2447468) (Scheme 2A), suggesting the formation and opening of a transient oxiranium intermediate. This illustrates the strong migratory aptitude of the 1,6‐oxygenated bridge, as previously observed with levoglucosan [46], which forced us to examine the alternative ring opening of epoxide **9**. Satisfyingly, treatment of **9** with NaN_3_ at 120°C for 48 h provided the expected C2‐azide **10c** (56% yield, 80% yield brsm). This regio‐ and stereo‐selectivity was further exemplified by introducing a fluorine atom with KHF_2_ at 210°C [47] to yield the fluorinated bicycle **10d**, albeit in modest 29% yield. Introduction of an allyl group was also successful using AllMgBr (10 equiv.) and CuI (5 equiv.) in THF [48] to afford the allyl derivative **10e** in 61% yield (Scheme 2B).

**SCHEME 2:**
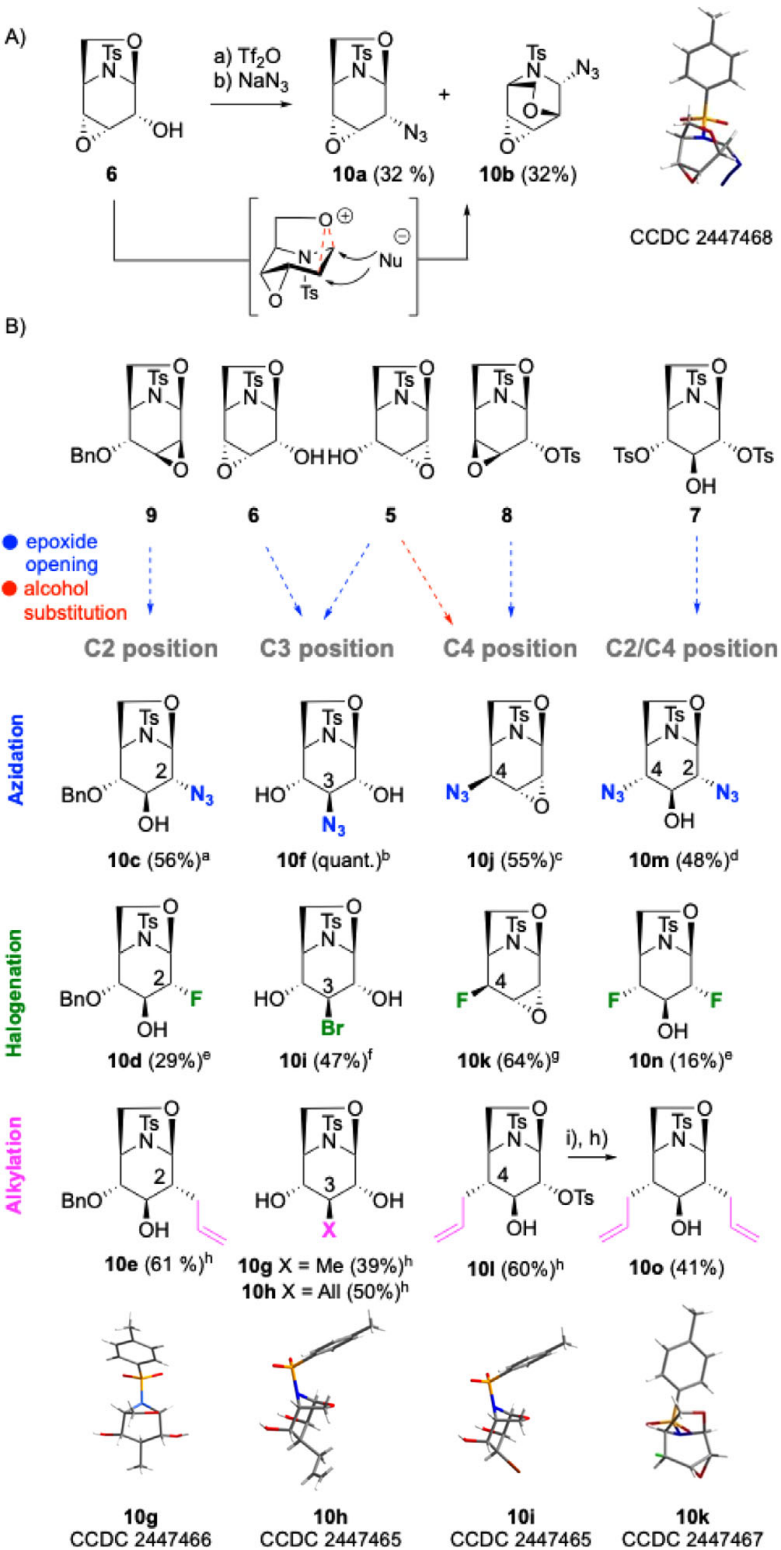
(A) Azidation of epoxide **6**; (B) One step C─O bond editing of C2, C3, and/or C4 positions from precursors **5–9;** (a) NaN_3_, DMF, 120°C, sealed tube, 48 h; (b) NaN_3_, DMF, 90°C, overnight; (c) Tf_2_O, CH_2_Cl_2_, 0°C then NaN_3_ DMF, rt, (d) NaN_3_, DMF, MW, 150 W, 150°C, 30 min; (e) KHF_2_, ethylene glycol, 170°C or 210°C, 2 h; (f) CH_2_=CHMgBr, Et_2_O, rt, overnight; (g) Tf_2_O, CH_2_Cl_2_, 0°C then TBAF.3H_2_O, CH_2_Cl_2_, rt, 48 h; (h) RMgBr, CuI, THF/H_2_O, rt; (i) MeONa, MeOH.

Diversification of the C3 position was investigated next from the mixture of epoxides **5** and **6**. Their azidation with NaN_3_ in DMF at 90°C for 24 h quantitatively provided C3‐azide **10f**. Alkylation (RMgX, CuI) was also successful as a methyl group (**10** **g**; CCDC 2447466) or an allyl group (**10** **h;** CCDC 2447465) were installed in 39% and 50% yields, respectively. However, when the reaction was performed with vinyl magnesium bromide to install a vinyl group as a CH_2_OH group precursor, only the bromohydrin **10i** (CCDC 2447465) was isolated in 47% yield (Scheme 2B) [49].

Diversification of the C4 position was then examined through OH‐4 group nucleophilic substitution. Unlike C2 position, migration of the 1,6‐oxygenated bridge was not observed, and treatment of hydroxyepoxide **5** with Tf_2_O in CH_2_Cl_2_ followed by NaN_3_ in DMF for 48 h furnished inverted azide **10j** in 55% yield. A fluorine atom was also successfully introduced at C4 position using TBAF.3H_2_O [28] to afford fluoro‐epoxide **10k** (64% yield, CCDC 2447467). In parallel, regioselective opening of epoxide **8** with AllMgBr (10 equiv.) and CuI (5 equiv.) produced C4‐allyl derivative **10l** in 60% yield (Scheme 2B).

Encouraged by these results, concomitant functionalization of the C2 and C4 positions to simultaneously and stereoselectively install the same function at two different positions on the piperidine ring was briefly investigated from ditosyl derivative **7**. While reaction with NaN_3_ in DMF at 80°C furnished a complex mixture of mono‐ and diazides, the same reaction performed under microwave irradiation cleanly afforded the diazide **10m** in 48% yield with complete retention of configuration [50]. Introduction of two fluorine atoms using KHF_2_ [28] was also successful, leading to difluoro derivative **10n**, albeit in modest 16% yield. Installation of two allyl groups was also achieved from monoallyl derivative **10l**, through formation of a 2,3‐endo‐epoxide under basic conditions followed by treatment with AllMgBr (10 equiv.) and CuI (5 equiv.) to furnish the diallyl derivative **10o** in 41% yield over two steps. Having achieved the regio‐ and stereoselective diversification of the *N*‐tosyl azalevoglucosan C2, C3, and C4 positions, C─O bond editing of the C1 position was then scrutinized.

Reductive ring opening of the bicyclic hemiaminal core should give access to piperidines that can be seen as modified iminoalditols, an important class of naturally occurring tetrahydroxylated piperidines [51] that serve as lead molecules for the treatment of diabetes, viral infections, inflammation, tumor growth, and lysosomal storage disorders [52]. To achieve this reaction, screening of a small range of Lewis acids (BF_3_.OEt_2_, TMSOTf, and TiCl_4_) in the presence of triethylsilane identified TiCl_4_ as the best Lewis acid in agreement with the literature [53, 54]. Treatment of hemiaminal **3** with Et_3_SiH and TiCl_4_ furnished partially protected iminoalditol **11a** in 66% yield, This reaction was also successful with hemiaminals **7**, **10f,** and **10l** to afford the corresponding tetrasubstituted *N*‐tosyl piperidines **11b‐d** in 44%–58% yield (Figure 2a). Accessing modified iminoalditols with a free endocyclic amine is also of interest to allow further amine coupling or to derivatize the nitrogen atom. Accordingly, treatment of hemiaminal **3** with LiAlH_4_ concomitantly reduced the hemiaminal bridge and removed the *N*‐Ts group to provide the piperidine **12a** in 78% yield. These conditions were then successfully applied to *N*‐tosyl azalevoglucosans **6**, **10o,** and **10f**, some of them being previously per‐*O*‐benzylated for purification issues, to yield the corresponding tri‐ and tetrasubstituted piperidines **12b–d** in 36%–51% yield after column chromatography (Figure 2b).

**FIGURE 2:**
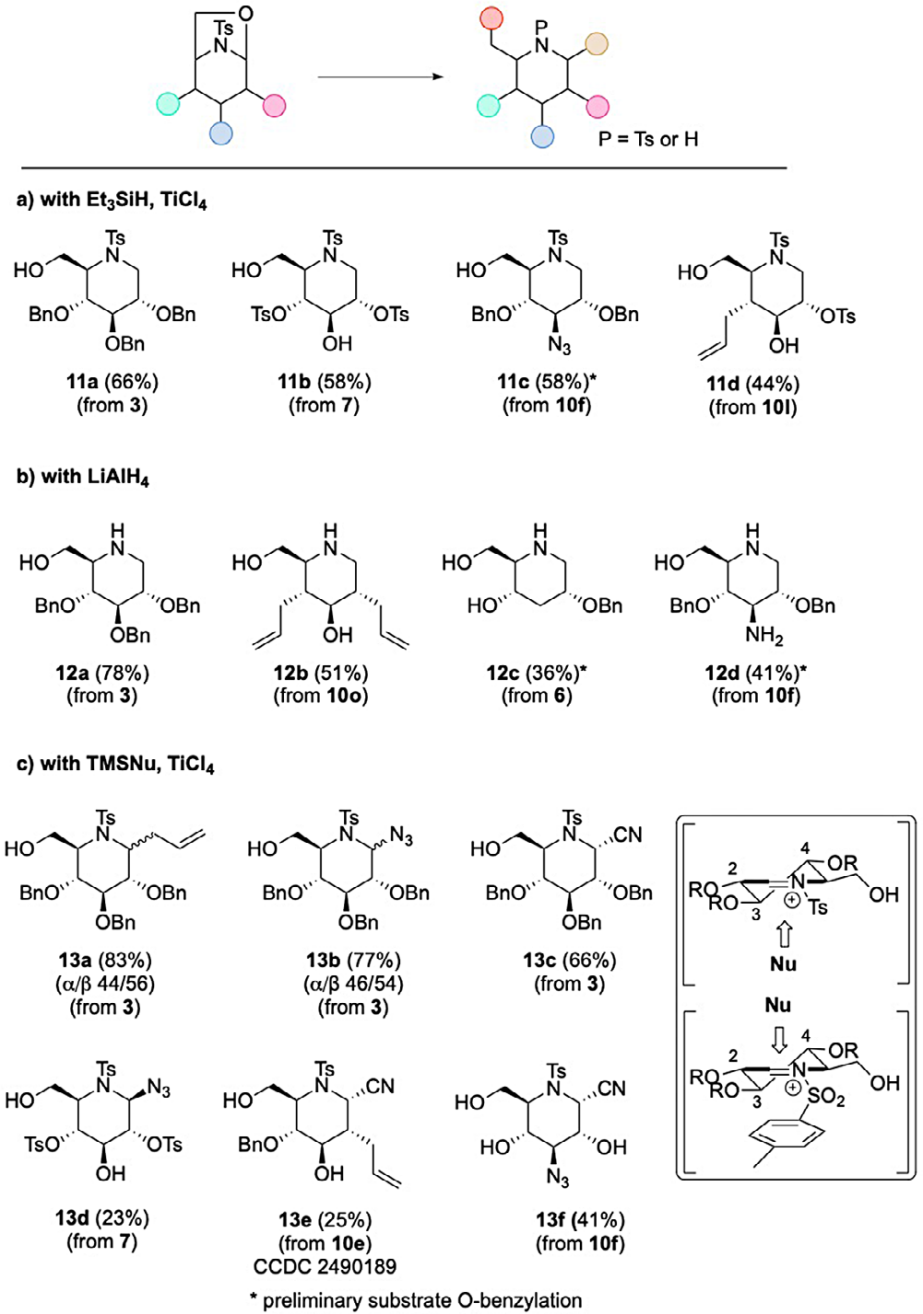
C1─O bond editing through hemiaminal ring opening. (a) Synthesis of tetrasubstituted *N*‐tosyl piperidines **11**. (b) Synthesis of tri‐ and tetra‐substituted NH‐free piperidines **12**. (c) Synthesis of pentasubstituted piperidines **13** and model to tentatively rationalize the stereochemical outcome of the reaction.

Capitalizing on the reductive cleavage of the *N*‐tosyl azalevoglucosan, introduction of an additional function at C1 position was then studied to reach pentasubstituted piperidines (Figure 2c). Fully *O*‐benzylated hemiaminal **3** was used as a model substrate. Its treatment with allyltrimethylsilane and TiCl_4_ afforded a separable mixture of α‐*C*‐allyl piperidine **13aα** (36%) and β‐*C*‐allyl piperidine **13aβ** (47%) (Figure 2c). Similarly, reaction with TMSN_3_ furnished α‐configured *C1*‐azido piperidine **13bα **(35% yield) and β‐configured **13bβ** (42% yield). The absence of stereocontrol in this reaction was rationalized as follows. An iminium intermediate adopting a ^4^H_3_ conformation can be invoked to account for the formation of the α−configured compounds [55]. The same iminium intermediate in which the aromatic ring of the *N*‐Ts group stacks below the piperidine ring could drive the formation of the β−configured compounds (Figure 2c) as suggested by the upcoming conformational analysis of the *N*‐tosyl pentasubstituted piperidines.

This reaction was also successful with TMSCN, exclusively providing the α‐*C1*‐cyano piperidine **13c** in 75% yield because of the anomeric effect [37]. The compatibility of this transformation with functionalized azalevoglucosan derivatives was also examined. Azidation of ditosyl derivative **7** produced azidopiperidine **13d** as a single β‐configured isomer, albeit in low 23% yield while cyanation of hemiaminals **10e** and **10f** furnished α‐configured piperidines **13e** (25% yield; CCDC 2490189) and **13f** (41% yield).

Finally, the iterative potential of this strategy was showcased with the aim to install a different functional group in a regio‐ and stereoselective manner on each piperidine's carbon in a stepwise manner. For our purpose, epoxide **6** was benzylated to afford epoxide **14** in 66% yield (Scheme 3a). Regioselective epoxide opening with sodium azide yielded azidoalcohol **15** (80% yield). Subsequent deoxyfluorination of the free alcohol with Tf_2_O and TBAF operated with inversion of configuration to afford hemiaminal **16** (71% yield) bearing three distinct functions at C2, C3, and C4 positions. Treatment of **16** with TMSAll and TiCl_4_ stereoselectively provided pentadifferentiated (*1S, 2R, 3R, 4R, 5R*) piperidine **17** in 39% yield. To further illustrate this stepwise approach, an aza analog of anhydro‐*N*‐acetyl muramic acid (anhNAM) [56], a 1,6‐anhydrosugar involved in peptidoglycan recycling in Gram‐negative bacteria, was targeted. Starting from azide **10c**, introduction of the lactate moiety at C3 position was achieved by treatment with the known triflate **18** [57] to afford ester **19** (CCDC 2447464) in 74% yield (Scheme 3b). Conversion of the azido group into an acetamide with triphenyl phosphine and aqueous ammonia followed by treatment with acetic anhydride gave acetamide **20** (53% yield). Final mild ester hydrolysis with 1 M LiOH in THF/dioxane (2/1) followed by hydrogenolysis (Pd(OH)2/C, H_2_) quantitatively afforded anhNAM analog **21**.

**SCHEME 3:**
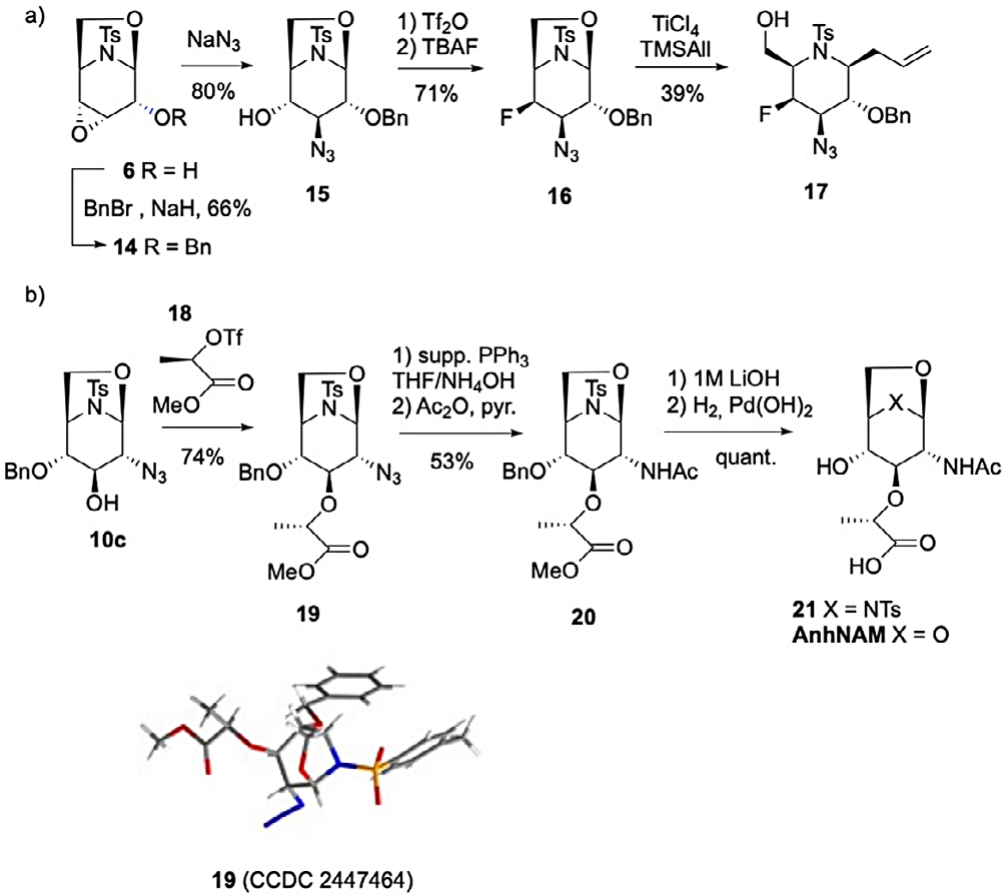
(a) Iterative, regio‐ and stereoselective introduction of functional groups on all the five contiguous carbons of a piperidine ring to produce pentadifferentiated piperidine **17**. (b) Synthesis of an aza analog **21** of anhydro‐N‐acetyl muramic acid and x‐ray structure of compound **19**.

The conformation of the *N*‐Ts pentasubstituted piperidines **13a‐f** and **17** was studied by NMR assisted by DFT calculations. In these molecules, the presence of the endocyclic sulfamido group forces the nitrogen atom to adopt a pyramidal–planar geometry, which in turn distorts the piperidine ring away from the canonical chair conformation. In addition, bulky substituents, electrostatic interactions, and solvent polarity further broaden the conformational landscape. These computed‐expected effects became particularly evident for azidopiperidine **13b**, for which both α‐ (**13b**α) and β‐ (**13b**β) conformers were independently examined. For the α‐epimer in CDCl_3_, the *J*_2,3_, *J*_3,4_, and *J*_4,5_ coupling constant values (9.0, 8.9, and 9.0 Hz, respectively) correspond to axial–axial relationships, characteristic of the canonical ^4^ *C*_1_ chair conformation. The *J*_1,2_ coupling constant (3.6 Hz) is typical of the equatorial–axial arrangement expected for the α‐anomer. Interestingly, the OH‐6 proton displays well defined coupling constants (4.1 Hz and 11.4 Hz) to the H‐6 protons, consistent with the existence of a hydrogen bond formed between OH‐6 and one oxygen atoms at the tosyl (Ts) group. The *J*_5,6_ and *J*_5,6’_ coupling values, both around 2.2 Hz, agree with a very predominant *gauche–gauche (gg)* arrangement around the hydroxymethyl group. NOE correlations between the tosyl aromatic protons (2/6‐Ts) and H‐1 (weak), H‐5 (weak), and H‐6 proS (strong) further support this arrangement and define the orientation of the aromatic ring relative to the pyranose framework (Figure 3a). For azidopiperidine **13b**β, the *J*_2,3_ and *J*_3,4_ values (both close to 8 Hz) suggest near axial–axial relationships, consistent with the benzyloxy substituents adopting pseudo‐equatorial positions. However, the *J*_1,2_ (4.4 Hz) and *J*_4,5_ (3.6 Hz) encode *gauche*‐like relationships (axial‐equatorial or related arrangements). A small, but measurable long‐range *J*_1,5_ coupling (0.4–0.5 Hz) indicates a W‐type arrangement, consistent with both protons displaying pseudo‐equatorial orientations. These findings point to a distortion of the chair conformation in the vicinity of the nitrogen atom. Unlike the α‐analogue, the OH‐6 proton appears as a broad signal, suggesting motion around the H6─O6 bond, consistent with the absence of stabilizing intramolecular hydrogen bonding. Accordingly, the *J*_5,6_ and *J*_5,6’_ coupling values, (5.1 and 5.6 Hz) reflect a dynamic equilibrium between the *gg* and *gt* conformations of the C5─C6 hydroxymethyl bond. The tosyl aromatic protons (2/6‐Ts) show NOEs with all H‐1, H‐5, and H‐3, confirming that the Ts group lies on the non‐polar face of the β‐conformer. Additional NOEs between H‐6/H‐6′ and both H‐2 and H‐4 define the spatial relationships around the C5─C6 region. Taken together, these data support a ^2^ *S*_N_ ring conformation as the predominant solution geometry of **13b**β (Figure 3b). Indeed, the expected (MSPIN‐calculated J‐values) for this ^2^ *S*_N_ geometry are in good agreement with the experimental values (see Table S1). For the other pentasubstituted piperidines **13a** and **13c‐f**, the scalar coupling analysis strongly suggests the existence of conformational equilibria, since the observed values are not consistent with a single conformation (see Table S2). Notably, the X‐ray crystallographic structure of **13e** shows a ^5^ *S*_1_ conformation in the solid state, while the analysis of the solution‐phase J coupling constants reveals the presence of a 60:40 conformational equilibrium between the crystallographic ^5^ *S*_1_ skew boat and the ^4^ *C*_1_ chair (see Table S3). For pentadifferentiated piperidine **17**, NOEs between H‐1 and H‐3, and between H‐6,6' and the allyl CH_2_ group confirmed the β‐configuration at C1 position. The observed *J*_1,2_, *J_2_*_,3_, *J*_3,4_ and *J*_4,5_ coupling constants values (<2, 6.9, <2, 5.6, respectively) are in good agreement with those obtained by DFT in Gaussian (0.7, 7.7, 1.9, 5.9 respectively), and all these findings are in full agreement with a ^1^ *S*_3_ ring conformation, flattened around the nitrogen atom (Figure 3c).

**FIGURE 3:**
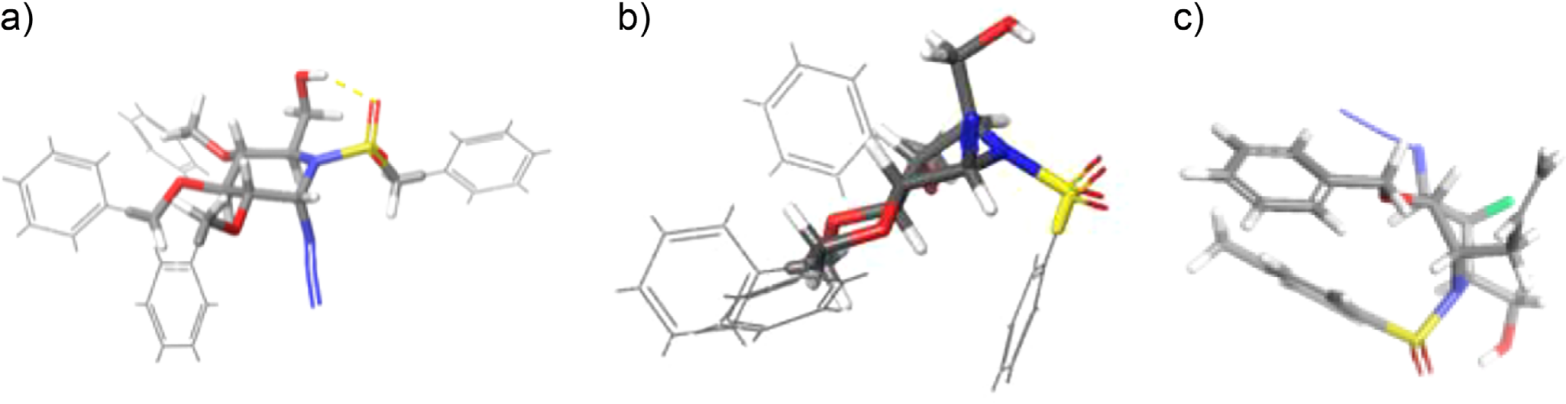
(a) Proposed major ^4^ *C*_1_ conformer for 1‐azidopiperidine **13b**α. (b) Proposed major ^2^ *S*_N_ conformer for 1‐azidopiperidine **13b**β. (c) Proposed major ^1^ *S*_3_ conformer for 1‐allylpiperidine **17**. The OPLS4 force field as implemented in Macromodel within the Maestro suite of programs was employed.

## 3 Conclusion

In conclusion, we have developed a robust synthesis of a *N*‐Ts azalevoglucosan derived from naturally occurring nojirimycin. As for levoglucosan, its 1,6‐anhydro bridge allows navigation on the piperidine ring to install, in a regio‐ and stereoselective manner, functional groups at C2, C3, and C4 positions. Moreover, the cleavage of its hemiaminal moiety gives access to piperidines with an additional function at C1 position. This approach is complementary to the aza‐Achmatowicz reaction [39]. The synthetic potential of this platform was illustrated by the iterative syntheses of an aza analog of anhydro‐muramic acid **21** and a pentadifferentiated (*1S, 2R, 3R, 4R, 5R*) 1‐allyl, 2‐benzyloxy‐, 3‐azido, 4‐fluoro, 5‐hydroxymethyl piperidine **17**. The platform reported herein should be useful in the glycomimetic field to access unprecedented iminosugar derivatives and in medicinal chemistry, where polysubstituted piperidine motifs are becoming increasingly popular.

## 4 Experimental Section

Experimental procedures and characterization data are available in the Supporting Information file. The authors have cited additional references within the Supporting Information [51, 52]. Deposition Number(s) 2447469 (for **4**), 2487464 (for **6**), 2447468 (for **10b**), 2447466 (for **10g**), 2447465 (for **10h**), 2447465 (for **10i**), 2447467 (for **10k**), 2490189 (for **13e**), 24474664 (for **19**) contain(s) the supplementary crystallographic data for this paper. These data are provided free of charge by the joint Cambridge Crystallographic Data Centre and Fachinformationszentrum Karlsruhe Access Structures service.

## Conflicts of Interest

The authors declare no conflicts of interest.

## Supporting information




**Supporting File 1**: chem71491‐sup‐0001‐SuppMat.docx

## Data Availability

The data that supports the findings of this study are available in the Supporting Information of this article.
