# Supplementary material for: N‐Sulfonyl Azalevoglucosan: A Versatile Platform for the Synthesis of Polysubstituted Piperidines
Source: Chemistry. 2026 Jul 28;32(40):e71491. doi: 10.1002/chem.71491 (PMC13716014; doi:10.1002/chem.71491)
Supplement: Supplementary file 1 [file CHEM-32-e71491-s001.docx]

***Supplementary Information***

***N*-Sulfonyl Azalevoglucosan: A Versatile Platform for the Synthesis of Polysubstituted Piperidines**

Dylan Yorga,^a^ Jérôme Marrot,^b^ Ana Poveda,^c^ Nicolas Auberger,^a^ Jesús Jiménez-Barbero,^c^ Jérôme Désiré,^a^* Yves Blériot^a^*

[a] Groupe Glycochimie, IC2MP-UMR CNRS 7285, Université de Poitiers, 4 rue Michel Brunet, 86073 Poitiers cedex 9, France

[b] Institut Lavoisier de Versailles, UVSQ, CNRS, UMR 8180, Université Paris-Saclay, 45 avenue des Etats-Unis, 78035 Versailles cedex, France

[c] ClC bioGUNE, Basque Research and Technology Alliance (BRTA), Bizkaia Technology Park, Building 800, 48160 Derio (Spain); Ikerbasque, Basque Foundation for Science, Maria Diaz de Haro 3, 48013 Bilbao, Bizkaia, Spain; Department of Organic & Inorganic Chemistry, Faculty of Science and Technology, University of the Basque Country, EHU-UPV Leioa, Spain; Centro de Investigacion Biomedica En Red de Enfermedades Respiratorias, Madrid, Spain

* E-mail: [yves.bleriot@univ-poitiers.fr](mailto:yves.bleriot@univ-poitiers.fr) [jerome.desire@univ-poitiers.fr](mailto:jerome.desire@univ-poitiers.fr)

**Table of contents**

1. General information	2
2. General procedures	2
3. Synthesis and characterization of new compounds	3
4. Crystallographic data for compounds **4**, **6**, **10b**, **10g**, **10h**, **10i**, **10k**, **13e, 19**	32
5. Copies of ^1^H, ^19^F and ^13^C NMR spectra of new compounds	45
6. NMR conformational analysis of piperidines **13a-f** and **17**	95
7. Copies of Infra-Red spectra	100

**1. General information**

All commercially available reagents were used as supplied without further purification. Petroleum ether (PE) refers to the 40-60°C boiling fraction. Air and water sensitive reactions were performed in “flame” dried glassware under Ar atmosphere. For reactions that require heating, Radleys® Heat-On block systems were used. TLC plates (MachereyNagel – Alugram® SIL G/UV254) were visualized under 254 nm UV light and/or by dipping the TLC plate into a solution of phosphomolybdic acid in ethanol (3 g/100 mL) followed by heating with a heat gun. Flash chromatography columns were performed using silica gel 60 (15-40 μm) or carried on a Combiflash® Rf automated apparatus (Teledyne-Isco) using columns specified in each protocol. When no column is mentioned, the product was purified on standard silica (Macherey-Nagel 60, 14-40 μm). IR data were obtained from an ALPHA II Compact FT-IR spectrometer and are given to the nearest 1 cm^−1^ with their intensity provided as follow: very strong (vs), strong (s), medium (m), weak (w), broad (br). Melting points were done on a Stuart automatic melting point SMP50 apparatus and are reported uncorrected. NMR experiments were recorded with a Bruker 400 Avance III Plus spectrometer at 400 MHz, 101 MHz, and 376 MHz for ^1^H, ^13^C and ^19^F nuclei respectively and with a Bruker Ultrashield^TM^ 500 Avance NEO spectrometer at 500 MHz, 125 MHz and 470 MHz for ^1^H, ^13^C and ^19^F nuclei respectively. Spectra were calibrated using the residual deuterated solvent peak and chemical shifts are expressed in part per million (ppm). NMR multiplicities are reported using the following abbreviations: b = broad, s = singlet, d = doublet, t = triplet, q = quadruplet, m = multiplet. Structural assignments were made with additional information from gCOSY, gHSQC, and gHMBC experiments. HRMS were obtained with a Q-TOF spectrometer from the Mass Spectrometry Service (IC2MP, UMR CNRS 7285-Poitiers University, France). Optical rotations were measured using a Modular Circular Polarimeter MCP100 (Anton Paar). Xray crystallography data were obtained from Institut Lavoisier de Versailles (UVSQ, CNRS UMR 8180, Paris-Saclay University, Versailles, France)*.* Numbering used for compounds names are given according to carbohydrates nomenclature, as below.

**2. General procedures**

**General procedure A for bicyclic hemiaminal debenzylation:** To a solution of bicyclic hemiaminal (1.0 eq.) in a mixture of MeOH/H_2_O (9:1, 0.75 M) under Ar atmosphere were added Pd/C 10% (1.2 eq), Pd black (0.12 eq) and ammonium formiate (15.0 eq). The reaction mixture was stirred for 4 h at 135°C. The reaction mixture was cooled to RT, filtered on decalite and concentrated to give the desired debenzylated bicyclic hemiaminal.

**General procedure B for activation of hydroxyl compound with triflic anhydride:** To a solution of hydroxyl compound (1.0 eq.) in dry DCM/dry Pyridine (6:1, 0.1 M), under Ar at 0°C, was added Tf_2_O (2.3 eq). The reaction mixture was stirred for 3-6 h at 0°C, then quenched with H_2_O and extracted twice with DCM. The combined organic layers were dried over MgSO_4_, filtered and concentrated to give the desired activated compound.

**General procedure C for epoxide ring opening with Grignard reagent:** To a solution of epoxide (1.0 eq.) in a mixture THF/Et_2_O (1:1, 0.1 M), under Ar at 0 °C, were added CuI (5.0 eq.) and RMgBr (10.0 eq.). The reaction mixture was stirred at room temperature until TLC shows consumption of the starting material. The reaction mixture was quenched at 0°C with a saturated solution of NH_4_Cl, then the resulting solution was extracted thrice with DCM. The combined organic layers were dried over MgSO_4_, filtered and concentrated. The crude was purified over a flash chromatography column.

**General procedure D for bicyclic hemiaminal ring opening with TiCl_4_ and TMS-Nu:** To a solution of bicyclic hemiaminal (1.0 eq.) in anhydrous CH_2_Cl_2_ (0.04 M) under Ar at -78°C, were added TMS-Nu (4.0 – 32.0 eq.) and dropwise TiCl_4_ 1 M (0.5 – 2.0 eq.). The reaction mixture was stirred at -78°C for 0.5 – 9 h until TLC shows consumption of the starting material. The reaction mixture was quenched with a saturated solution of NaHCO_3_, then the resulting solution was extracted thrice with DCM. The combined organic layers were dried over MgSO_4_, filtered and concentrated. The crude was purified over a flash chromatography column.

**General procedure E for bicyclic hemiaminal ring opening with LiAlH_4_:** To a suspension of LiAlH_4_ (6.0 eq.) in anhydrous THF, under Ar at RT, was added dropwise a solution of bicyclic hemiaminal (1.0 eq) in anhydrous THF. The reaction mixture was then stirred at 85°C for 2 to 6 h until TLC showed total consumption of the starting material. The reaction mixture was cooled down to RT, quenched with water and extracted four times with EtOAc. The combined organic layers were dried over MgSO_4_, filtered and concentrated. The crude was purified over a flash chromatography column.

**3. Synthesis and characterization of new compounds**

###### **6-*O*-Acetyl-2,3,4-tri-*O*-benzyl-*N*-tosyl-D-glucono-δ-lactam 2**

To a solution of lactam **1’** (4.9 g, 7.2 mmol, 1.0 eq) in dry DCM under Ar atm. at 0°C were added Ac_2_O (9.5 mL, 100.3 mmol, 14.0 eq) and FeCl_3_ (2.3 g, 14.3 mmol, 2.0 eq). After 20 min at 0°C, TLC showed total consumption of the starting material. The reaction mixture was neutralized with a 1:1 solution of saturated NaHCO_3_/ice (1 L). The aqueous layer was extracted with DCM (4x300 mL). The combined organic layers were dried over MgSO_4_ and concentrated under reduced pressure. The residue was purified over a flash chromatography column (RediSep® 80 g, PE/EtOAc 90:10 to 80:20) to afford **2** (3.91 g, 86 %) as a colorless oil. **R*_f_* =** 0.22 (PE/EtOAc 85:15); $\left[ \boldsymbol{\alpha} \right]_{\mathbf{D}}^{\mathbf{23}}$ **=** +44.1 (*c* 1.0, CHCl_3_); **^1^H NMR (400 MHz, CDCl_3_): δ** 7.82 (d, 2H, *J* = 8.3 Hz, H-Ar), 7.38 (dd, 2H, *J* = 7.6, 1.4 Hz, H-Ar), 7.35 - 7.28 (m, 9H*,* H-Ar), 7.23 - 7.18 (m, 2H, H-Ar), 7.17 - 7.08 (m, 2H, H-Ar), 6.98 (d, 2H, *J* = 8.2 Hz, H-Ar), 5.06 (d, 1H, *J* = 11.5 Hz, OC*H*HPh), 4.95 (td, 1H, *J* = 6.3, 2.0 Hz, H-5), 4.60 (d, 1H, *J* = 11.5 Hz, OC*H*HPh), 4.58 (d, 1H, *J* = 11.7 Hz, OC*H*HPh), 4.51 (d, 1H, *J* = 11.7 Hz, OC*H*HPh), 4.45 (dd, 1H, *J* = 11.8, 8.6 Hz, H-6’), 4.34 (m, 4H, OC*H*_2_Ph, H-6, H-2), 3.80 - 3.74 (m, 2H, H-3, H-4), 2.30 (s, 3H, CH_3_-Ts), 2.08 (s, 3H, CH_3_-CO); **^13^C NMR (100 MHz, CDCl_3_): δ** 170.9 (C^IV^, C=O), 168.4 (C^IV^, C_1_=O), 144.7, 137.3, 136.6, 135.5 (C^IV^, C-Ar), 129.2, 129.1, 128.6, 128.6, 128.5, 128.4, 128.2, 128.2, 128.1, 128.0 (CH-Ar), 81.3 (C_3_ or C_4_), 79.8 (C_2_), 75.1 (C_3_ or C_4_), 74.2 (O*C*H_2_Ph), 72.8 (O*C*H_2_Ph), 71.2 (O*C*H_2_Ph), 62.0 (C_6_), 59.3 (C_5_), 21.7 (CH_3_-Ts), 21.0 (CH_3_-CO); **IR (cm^-1^)**: 2900 (w), 1720 (s), 1351 (s); **HRMS (ESI) m/z =** [M+H]^+^ calcd for C_36_H_38_NO_8_S: 644.2313; found 644.2329.

###### **1,6-Anhydro-2,3,4-tri-*O*-benzyl-*N*-tosyl-β-D-glucopiperidinose 3**

To a solution of lactam **2** (3.8 g, 6.0 mmol, 1.0 eq) in dry DCM (60 mL) under Ar atm. at -78°C was added dropwise DIBAL-H (1M, 18 mL, 18.0 mmol, 3.0 eq). The reaction was stirred at -78°C for 10 min, when a TLC showed total consumption of the starting material. The reaction mixture was neutralized by slow addition of MeOH (25 mL), then H_2_O (50 mL) and HCl 1M (50 mL) were added. The reaction mixture was extracted with DCM (3x100 mL). The combined organic layers were dried over MgSO_4_ and concentrated under reduced pressure. The crude product (3.0 g) was directly engaged in the next step.

The crude residue (3.0 g) was dissolved in dry DCE (60 mL) under Ar atm. and CSA (697 mg, 3.0 mmol, 0.5 eq) was added at RT. The mixture was stirred overnight at RT when TLC showed total consumption of the starting material. H_2_O (100 mL) was added, and the mixture was then extracted with DCM (3x300 mL). The combined organic layers were dried over MgSO_4_ and concentrated under reduced pressure. The residue was purified over a flash chromatography column (RediSep® 40 g, PE/EtOAc 90:10 to 75:25) to afford hemiaminal **3** (2.7 g, 77 %, 2 steps) as a white solid. **R*_f_* =** 0.49 (PE/AcOEt 80:20); mp: 82.6 °C; $\left[ \boldsymbol{\alpha} \right]_{\mathbf{D}}^{\mathbf{20}}$ **=** -18.2 (*c* 1.0, CHCl_3_); **^1^H NMR (400 MHz, CDCl_3_): δ** 7.85 (d, 2H, *J* = 8.3 Hz, H-Ar), 7.36 - 7.27 (m, 10H, H-Ar), 7.25 - 7.17 (m, 5H, H-Ar), 7.15 (d, 2H, *J* = 8.3 Hz, H-Ar), 5.75 (br.s, 1H, H-1), 4.56 (d, 1H, *J* = 12.1 Hz, OC*H*HPh), 4.49 (d, 1H, *J* = 12.1 Hz, OC*H*HPh), 4.48 - 4.38 (m, 5H, 2xOC*H*_2_Ph and H-5), 3.82 (d, 1H, *J* = 7.5 Hz, H-6’), 3.58 (br.s, 2H, H-2 and H-3), 3.46 (br.s, 1H, H-4), 3.43 (dd, 1H, *J* = 7.5, 6.0 Hz, H-6), 2.36 (s, 3H, CH_3_-Ts); **^13^C NMR (100 MHz, CDCl_3_): δ** 143.9, 137.8, 137.8, 137.8, 136.5 (C^IV^, C-Ar), 129.5, 128.6, 128.5, 128.5, 128.1, 128.0, 127.9, 127.9, 127.8, 127.8 (CH-Ar), 86.9 (C_1_), 78.4 (C_4_), 78.0 (C_2_ or C_3_), 77.7 (C_2_ or C_3_), 72.2 (O*C*H_2_Ph), 71.4 (O*C*H_2_Ph), 70.6 (O*C*H_2_Ph), 66.1 (C_6_), 57.4 (C_5_), 21.7 (CH_3_-Ts); **HRMS (ESI) m/z =** [M+Na]^+^ calcd for C_34_H_35_NNaO_6_S : 608.2077; found 608.2104.

###### **1,6-Anhydro-*N*-tosyl-β-D-glucopiperidinose 4**

Bicyclic hemiaminal **3** (0.50 g, 0.85 mmol, 1.0 eq.) was deprotected following general **procedure A** to give triol **4** (0.27 g, quant.) as a white solid without purification. **R*_f_* =** 0.18 (PE/AcOEt 20:80); mp: 198-200°C (decomposition); $\left[ \boldsymbol{\alpha} \right]_{\mathbf{D}}^{\mathbf{20}}$ **=** +82.6 (*c* 0.5, CHCl_3_); **^1^H NMR (400 MHz, CD_3_OD): δ** 7.82 (d, 2H, *J* = 8.2 Hz, H-Ar), 7.39 (d, 2H, *J* = 8.2 Hz, H-Ar), 5.52 (d, 1H, *J* = 2.4 Hz, H-1), 4.25 (d, 1H, *J* = 6.9 Hz, *J* = 1.8 Hz, H-5), 3.78 (d, 1H, *J* = 7.9 Hz, H-6’), 3.56 (m, 3H, H-2, H-3 and H-4), 2.79 (dd, *J* = 7.9 Hz, *J* = 5.7 Hz, 1H), 2.44 (s, 3H, CH_3_-Ts); **^13^C NMR (100 MHz, CD_3_OD): δ** 146.1, 137.1 (C^IV^, C-Ar), 130.9, 129.1 (CH-Ar), 91.3 (C_1_), 76.0 (C_2_ or C_3_ or C_4_), 74.4 (C_2_ or C_3_ or C_4_), 74.0 (C_2_ or C_3_ or C_4_), 66.1 (C_6_), 62.3 (C_5_), 21.5 (CH_3_-Ts); **HRMS (ESI) m/z =** [M+Na]^+^ calcd for C_13_H_17_NNaO_6_S 338.0669; found 338.0678; **X-ray crystallography**: CCDC 2447469 (see p 32).

###### **1,6:3,4-Dianhydro-*N*-tosyl-β-D-allopiperidinose 6 and 1,6:2,3-dianhydro-*N*-tosyl-β-D-allopiperidinose 5**

To a solution of triol **4** (40 mg, 0.13 mmol, 1.0 eq) in dry THF (1.58 mL) under Ar atm. at 0°C were added PMe_3_ (1M/THF, 511 μL, 0.51 mmol, 4.0 eq) and TMAD (88 mg, 0.51 mmol, 4.0 eq). The reaction mixture was stirred for 3 h at 90°C, then cooled down to RT and concentrated under reduced pressure. The residue was diluted in AcOEt/H_2_O (10/10 mL) and the aqueous layer was extracted with AcOEt (3x15 mL). The combined organic layers were dried over MgSO_4_ and concentrated under reduced pressure. The residue was purified over a flash chromatography column (PE/EtOAc 90:10 to 60:40) to afford epoxides **6** (8 mg, 21 %) and **5** (26 mg, 68%) as white solids.

**Data for compound 6: R*_f_* =** 0.69 (PE/AcOEt 20:80); $\left[ \boldsymbol{\alpha} \right]_{\mathbf{D}}^{\mathbf{25}}$ **=** -83.0 (*c* 0.4, CHCl_3_); **^1^H NMR (400 MHz, CDCl_3_): δ** 7.76 (d, 2H, *J* = 8.2 Hz, H-Ar), 7.33 (d, 2H, *J* = 8.2 Hz, H-Ar), 5.34 (s, 1H, H-1), 4.68 (t, 1H, *J* = 3.6 Hz, H-5), 3.81 – 3.70 (d, 2H, H-6’, H-2), 3.37 (t, 1H, *J* = 3.5 Hz, H-4), 3.27 (dd, 1H, *J* = 5.6, 2.6 Hz, H-3), 3.21 (dd, 1H, *J* = 7.9, 4.3 Hz, H-6), 2.44 (s, 3H, CH_3_-Ts), 2.35 (d, 1H, *J* = 12.2 Hz, C_2_-O*H*); **^13^C NMR (100 MHz, CDCl_3_): δ** 144.9, 135.8 (C^IV^, C-Ar), 130.2, 127.6 (CH-Ar), 88.7 (C_1_), 66.6 (C_6_), 66.5 (C_2_), 54.5 (C_5_), 52.2 (C_4_), 49.4 (C_3_), 21.8 (CH_3_-Ts); **HRMS (ESI) m/z =** [M+H]^+^ calcd for C_13_H_16_O_5_NS 298.0744; found 298.0736. **X-ray crystallography**: CCDC 2487464 (see p 33)

**Data for compound 5: R*_f_* =** 0.64 (PE/AcOEt 20:80); $\left[ \boldsymbol{\alpha} \right]_{\mathbf{D}}^{\mathbf{25}}$ **=** +7.0 (*c* 0.2, CHCl_3_); **^1^H NMR (400 MHz, CDCl_3_): δ** 7.76 (d, 2H, *J* = 8.2 Hz, H-Ar), 7.32 (d, 2H, *J* = 8.2 Hz, H-Ar), 6.01 (d, 1H, *J* = 2.4 Hz, H-1), 4.08 (dd, 1H, *J* = 6.5, 1.6 Hz, H-5), 3.70 (ddd, 1H, *J* = 12.2, 4.7, 2.2 Hz, H-4), 3.42 (dd, 1H, *J* = 8.6, 1.7 Hz, H-6’), 3.36 – 3.29 (m, 2H, H-3, H-2), 3.22 (dd, 1H, *J* = 8.5, 6.7 Hz, H-6), 2.75 (d, 1H, *J* = 12.2 Hz, C_4_-O*H*), 2.44 (s, 3H, CH_3_-Ts); **^13^C NMR (100 MHz, CDCl_3_): δ** 144.9, 135.3 (C^IV^, C-Ar), 130.0, 127.9 (CH-Ar), 85.8 (C_1_), 66.8 (C_4_), 65.2 (C_6_), 61.6 (C_5_), 50.9 (C_2_ or C_3_), 50.4 (C_2_ or C_3_), 21.8 (CH_3_-Ts); **HRMS (ESI) m/z =** [M+H]^+^ calcd for C_13_H_16_O_5_NS 298.0744; found 298.0725.

###### **1,6-Anhydro-2,4-di-*O*-tosyl-*N*-tosyl-β-D-glucopiperidinose 7**

To a solution of triol **4** (400 mg, 1.27 mmol, 1.0 eq.) in dry pyridine (12.7 mL) under Ar were added DMAP (31 mg, 0.25 mmol, 0.2 eq.) and TsCl (1.45 g, 7.61 mmol, 6.0 eq.) at 0°C. The mixture was stirred overnight at RT, then cooled to 0°C and 1M HCl (15 mL) was added. The aqueous layer was extracted with AcOEt (3x50 mL) then the combined organic layers were washed with sat. NaHCO_3_ (50 mL) and brine (50 mL). The organic layer was dried over MgSO_4_, evaporated and co-evaporated with toluene. The residue was purified over a flash chromatography column (PE/EtOAc 90:10 to 60:40) to afford tosylate **7** (650 mg, 82 %) as a white solid. **R*_f_* =** 0.67 (PE/AcOEt 50:50); mp: 136.8°C; $\left[ \boldsymbol{\alpha} \right]_{\mathbf{D}}^{\mathbf{20}}$ **=** -35.2 (*c* 0.5, CHCl_3_); **^1^H NMR (400 MHz, CDCl_3_): δ** 7.74 – 7.68 (m, 4H, H-Ar), 7.64 (d, 2H, *J* = 8.3 Hz, H-Ar), 7.38 – 7.31 (m, 4H, H-Ar), 7.22 (d, 2H, *J* = 8.1 Hz, H-Ar), 5.54 (d, 1H, *J* = 2.5 Hz, H-1), 4.40 - 4.35 (m, 2H, H-2, H-4), 4.35 – 4.32 (m, 1H, H-5), 3.94 – 3.88 (m, 2H, H-6’, H-3), 3.51 (dd, 1H, *J* = 8.4, 5.4 Hz, H-6), 2.65 (d, 1H, *J* = 6.2 Hz, 3-O*H*), 2.47 (s, 6H, 2xCH_3_-Ts), 2.42 (s, 3H, CH_3_-Ts); **^13^C NMR (100 MHz, CDCl_3_): δ** 145.7, 145.6, 144.7, 135.4, 132.9, 132.6 (C^IV^, C-Ar), 130.3, 130.2, 129.8, 128.1, 128.1, 128.0 (CH-Ar), 86.3 (C_1_), 78.6 (C_2_ and C_4_), 70.8 (C_3_), 66.6 (C_6_), 58.5 (C_5_), 21.9 (2x CH_3_-Ts), 21.8 (CH_3_-Ts); **HRMS (ESI) m/z =** [M+H]^+^ calcd for C_27_H_30_NO_10_S_3_ 624.7140; found 624.1003.

###### **1,6:3,4-Dianhydro-2-*O*-tosyl-*N*-tosyl-β-D-galactopiperidinose 8**

To a solution of tosylate **7** (640 mg, 1.03 mmol, 1.0 eq) in dry MeOH (10.3 mL) under Ar atm. at 0°C was added Na° (236 mg, 10.3 mmol, 10.0 eq). The reaction was stirred at 0°C for 1 h 30, when TLC showed total consumption of the starting material. The reaction mixture was neutralized with addition of Amberlite IR 120H resin. After filtration and concentration under reduced pressure, the crude was diluted in DCM/H_2_O and extracted with DCM (3x20 mL). The combined organic layers were dried over MgSO_4_ and concentrated under reduced pressure to afford epoxide **8** (460 mg, quant.) as a white solid. **R*_f_* =** 0.50 (PE/AcOEt 60:40); $\left[ \boldsymbol{\alpha} \right]_{\mathbf{D}}^{\mathbf{20}}$ **=** -222.0 (*c* 0.1, CHCl_3_); **^1^H NMR (400 MHz, CDCl_3_): δ** 7.79 (d, 2H, *J* = 8.3 Hz, H-Ar), 7.74 (d, 2H, *J* = 8.3 Hz, H-Ar), 7.37 (d, 2H, *J* = 8.1 Hz, H-Ar), 7.32 (d, 2H, *J* = 8.1 Hz, H-Ar), 5.29 (s, 1H, H-1), 4.62 (t, 1H, *J* = 5.4 Hz, H-5), 4.56 (d, 1H, *J* = 1.6 Hz, H-2), 3.78 (d, 1H, *J* = 7.2 Hz, H-6’), 3.68 (dd, 1H, *J* = 5.8, 4.0 Hz, H-4), 3.10 (dd, 1H, *J* = 3.7, 1.2 Hz, H-3), 2.99 (dd, 1H, *J* = 7.2, 4.8 Hz, H-6), 2.47 (s, 3H, CH_3_-Ts), 2.45 (s, 3H, CH_3_-Ts); **^13^C NMR (100 MHz, CDCl_3_): δ** 145.8, 145.1, 135.2, 132.7 (C^IV^, C-Ar), 130.3, 130.1, 128.2, 128.0 (CH-Ar), 86.2 (C_1_), 72.4 (C_2_), 65.6 (C_6_), 54.8 (C_5_), 54.5 (C_4_), 49.0 (C_3_), 21.9 (CH_3_-Ts), 21.8 (CH_3_-Ts); **HRMS (ESI) m/z =** [M+H]^+^ calcd for C_20_H_22_NO_7_S_2_ 452.0832; found 452.0818.

###### **1,6:2,3-Dianhydro-4-*O*-benzyl-*N*-tosyl-β-D-mannopiperidinose 9**

To a solution of epoxide **8** (680 mg, 1.50 mmol, 1.0 eq) in dry toluene (15 mL, 0.1 M) under Ar atm. at RT were added benzyl alcohol (468 μL, 4.50 mmol, 3.0 eq) and BF_3_.OEt_2_ (190 μL, 1.50 mmol, 1.0 eq). The reaction mixture was stirred at 50°C for 7 h. The reaction mixture was cooled down to RT, quenched with sat. NaHCO_3_ (10 mL) and the aqueous layer was extracted with AcOEt (4x15 mL). The combined organic layers were washed with H_2_O (20 mL), dried over MgSO_4_ and concentrated under reduced pressure. The residue was purified over a chromatography column (PE/EtOAc 70:30 to 50:50) to afford **1,6-anhydro-4-*O*-benzyl-2-*O*-tosyl-*N*-tosyl-β-D-glucopiperidinose** (420 mg, 50 %). **R*_f_* =** 0.17 (PE/AcOEt 70:30); $\left[ \boldsymbol{\alpha} \right]_{\mathbf{D}}^{\mathbf{20}}$ **=** -4.0 (*c* 0.3, CHCl_3_); **^1^H NMR (400 MHz, CDCl_3_): δ** 7.76 (d, 2H, *J* = 8.2 Hz, H-Ar), 7.71 (d, 2H, *J* = 8.2 Hz, H-Ar), 7.35 – 7.27 (m, 5H, H-Ar), 7.20 – 7.12 (m, 4H, H-Ar), 5.54 (d, 1H, *J* = 2.2 Hz, H-1), 4.47 (s, 2H, OC*H_2_*Ph), 4.42 (d, 1H, *J* = 5.2 Hz, H-5), 4.37 (t, 1H, *J* = 2.1 Hz, H-2), 3.96 (s, 1H, H-3), 3.84 (d, 1H, *J* = 8.0 Hz, H-6’), 3.51 (dd, 1H, *J* = 8.0, 5.2 Hz, H-6), 3.44 (s, 1H, H-4), 2.51 (s, 1H, C_3_-O*H*), 2.45 (s, 3H, CH_3_-Ts), 2.37 (s, 3H, CH_3_-Ts); **^13^C NMR (100 MHz, CDCl_3_): δ** 145.4, 144.3, 137.3, 135.8, 132.8 (C^IV^, C-Ar), 130.0, 129.5, 128.4, 128.0, 127.9, 127.8, 127.6 (CH-Ar), 86.5 (C_1_), 79.4 (C_4_), 78.7 (C_2_), 71.2 (C_3_), 70.9 (O*C*H_2_Ph), 66.8 (C_6_), 57.6 (C_5_), 21.8 (CH_3_-Ts), 21.7 (CH_3_-Ts); **HRMS (ESI) m/z =** [M+H]^+^ calcd for C_27_H_30_O_8_NS_2_ 560.1407; found 560.1403.

To a suspension of **1,6-anhydro-4-*O*-benzyl-2-*O*-tosyl-*N*-tosyl-β-D-glucopiperidinose** (350 mg, 0.625 mmol, 1.0 eq.) in dry MeOH (7 mL), at 0°C under Ar, were added dry toluene (0.6 mL, to obtain a clear solution) and sodium (144 mg, 6.25 mmol, 10.0 eq.). The solution was stirred at RT under Ar for 4h, then diluted with DCM (30 mL), and quenched with water (30 mL) and H_2_SO_4_ (1M, 3 mL). The aqueous phase was extracted with DCM (3 x 30 mL), the combined organic layers were dried over MgSO_4_ and concentrated under reduced pressure to afford epoxide **9** (242 mg, quant., 50% over 2 steps) as a colorless oil. **R*_f_* =** 0.67 (PE/AcOEt 60:40); $\left[ \boldsymbol{\alpha} \right]_{\mathbf{D}}^{\mathbf{20}}$ **=** +6.4 (*c* 1.0, CHCl_3_); **^1^H NMR (400 MHz, CDCl_3_): δ** 7.72 (d, 2H, *J* = 8.3 Hz, H-Ar), 7.29 - 7.18 (m, 7H, H-Ar), 5.88 (d, 1H, *J* = 4.3 Hz, H-1), 4.59 (d, 1H, *J* = 11.6 Hz, OCH*H*Ph), 4.50 (d, 1H, *J* = 11.6 Hz, OCH*H*Ph), 4.14 (d, 1H, *J* = 6.7 Hz, H-5), 3.67 (d, 1H, *J* = 0.8 Hz, H-4), 3.52 (t, 1H, *J* = 4.3 Hz, H-2), 3.37 (dd, 1H, *J* = 7.7, 1.5 Hz, H-6’), 3.04 (dd, 1H, *J* = 3.6, 0.9 Hz, H-3), 2.99 (t, 1H, *J* = 7.1 Hz, H-6), 2.34 (s, 3H, CH_3_-Ts); **^13^C NMR (100 MHz, CDCl_3_): δ** 144.5, 136.9, 135.1 (C^IV^, C-Ar), 129.7, 128.5, 128.0, 127.8 (CH-Ar), 84.4 (C_1_), 74.6 (C_4_), 71.6 (O*C*H_2_Ph), 65.8 (C_6_), 55.4 (C_5_), 55.2 (C_2_), 49.3 (C_3_), 21.6 (CH_3_-Ts); **HRMS (ESI) m/z =** [M+H]^+^ calcd for C_20_H_22_NO_5_S 388.1213; found 388.1197.

###### **1,6:3,4-Dianhydro-2-azido-2-deoxy-*N*-tosyl-β-D-allopiperidinose 10a and 2,6:3,4-Dianhydro-1-azido-1-deoxy-*N*-tosyl-β-D-altropiperidinose 10b**

Epoxide **6** (20 mg, 0.07 mmol, 1.0 eq.) was treated with Tf_2_O (25.4 μL, 0.16 mmol, 2.3 eq.) following **procedure B** to give the expected triflate (31 mg, quant.) as a yellow oil which was directly used in the following step without purification. To a solution of this triflate in dry DMF (673 μL, 0.1M) under Ar atm. at RT was added NaN_3_ (26 mg, 0.40 mmol, 6.0 eq). The reaction mixture was stirred at RT for 72 h, when TLC showed total consumption of the triflate. The reaction mixture was concentrated under reduced pressure. The residue was purified over a flash chromatography column (RediSep® 4 g, PE/EtOAc 90:10 to 60:40) to afford azides **10a** (7 mg, 32 %) and **10b** (7 mg, 32 %) as white solids.

**Data for azide 10a: R*_f_* =** 0.64 (PE/AcOEt 70:30); $\left[ \boldsymbol{\alpha} \right]_{\mathbf{D}}^{\mathbf{20}}$ **=** -22.0 (*c* 0.2, CHCl_3_); **^1^H NMR (400 MHz, CDCl_3_): δ** 7.76 (d, 2H, *J* = 8.2 Hz, H-Ar), 7.34 (d, 2H, *J* = 8.2 Hz, H-Ar), 5.62 (t, 1H, *J* = 2.7 Hz, H-1), 4.61 (t, 1H, *J* = 3.6 Hz, H-5), 3.88 (d, 1H, *J* = 8.1 Hz, H-6’), 3.51 (d, 1H, *J* = 2.9 Hz, H-2), 3.32 – 3.27 (m, 2H, H-3, H-6), 3.07 (dd, 1H, *J* = 3.7, 2.3 Hz, H-4), 2.44 (s, 3H, CH_3_-Ts); **^13^C NMR (100 MHz, CDCl_3_): δ** 145.1, 135.6 (C^IV^, C-Ar), 130.2, 127.7 (CH-Ar), 86.1 (C_1_), 68.3 (C_6_), 58.0 (C_2_), 54.3 (C_5_), 52.1 (C_3_), 49.1 (C_4_), 21.8 (CH_3_-Ts); **HRMS (ESI) m/z =** [M+H]^+^ calcd for C_13_H_15_O_4_N_4_S 323.0808; found 323.0796.

**Data for azide 10b: R*_f_* =** 0.34 (EP/AcOEt 70:30); $\left[ \boldsymbol{\alpha} \right]_{\mathbf{D}}^{\mathbf{20}}$ **=** +4.0 (*c* 0.3, CHCl_3_); **^1^H NMR (500 MHz, CDCl_3_): δ** 7.84 (d, 2H, *J* = 8.4 Hz, H-Ar), 7.36 (d, 2H, *J* = 8.4 Hz, H-Ar), 5.22 (d, 1H, *J* = 3.6 Hz, H-1), 4.37 (dd, 1H, *J* = 4.6, 2.3 Hz, H-5), 4.30 (t, 1H, *J* = 3.9 Hz, H-2), 3.73 (dd, 1H, *J* = 9.4, 2.4 Hz, H-6’), 3.57 (td, 1H, *J* = 4.4, 1.1 Hz, H-3), 3.52 – 3.49 (m, 2H, H-6 and H-4), 2.45 (s, 3H, CH_3_); **^13^C NMR (125 MHz, CDCl_3_): δ** 144.8, 136.3 (C^IV^, C-Ar), 130.2, 127.7 (CH-Ar), 72.8 (C_1_), 70.5 (C_2_), 62.8 (C_6_), 49.7 (C_5_), 48.2 (C_4_), 47.2 (C_3_), 21.8 (CH_3_); **HRMS (ESI) m/z =** [M+H]^+^ calcd for C_13_H_15_O_4_N_4_S 323.0808; found 323.0799; **X-ray crystallography**: CCDC 2447468 (see p 35).

###### **1,6-Anhydro-2-azido-4-*O*-benzyl-2-deoxy-*N*-tosyl-β-D-glucopiperidinose 10c**

To a solution of epoxide **9** (116 mg, 0.299 mmol, 1.0 eq.) in a mixture of DMF and water (3 mL/300 μL), in a sealed tube, was added NaN_3_ (116 mg, 1.79 mmol, 6.0 eq.). The suspension was stirred at 120°C for 70h, then cooled down to RT, diluted with water (25 mL) and extracted with Et_2_O (3 x 20 mL). The combined organic layers were dried over MgSO_4_ and concentrated under reduced pressure. The crude was purified over a chromatography column (PE/EtOAc 70:30 then 60:40) to afford azide **10c** (72 mg, 56 %) a colorless an oil. **R*_f_* =** 0.37 (PE/AcOEt 50:50); $\left[ \boldsymbol{\alpha} \right]_{\mathbf{D}}^{\mathbf{20}}$ **=** -18.7 (*c* 1.0, CHCl_3_); **^1^H NMR (400 MHz, CDCl_3_): δ** 7.76 (d, 2H, *J* = 8.2 Hz, H-Ar), 7.27 - 7.16 (m, 7H, H-Ar), 5.65 (d, 1H, *J* = 2.2 Hz, H-1), 4.50 (d, 1H, *J* = 11.9 Hz, OCH*H*Ph), 4.42 (d, 1H, *J* = 11.8 Hz, OCH*H*Ph), 4.32 (d, 1H, *J* = 4.2 Hz, H-5), 3.81 (brs, 1H, H-3), 3.77 (d, 1H, *J* = 7.9 Hz, H-6’), 3.39 (t, 1H, *J* = 2.3 Hz, H-4), 3.34 – 3.32 (m, 2H, H-6, H-2), 2.34 (s, 3H, CH_3_-Ts); **^13^C NMR (100 MHz, CDCl_3_): δ** 144.6, 137.0, 135.4 (C^IV^, C-Ar), 129.7, 128.5, 128.0, 127.9, 127.7 (CH-Ar), 87.8 (C_1_), 79.6 (C_4_), 71.6 (C_3_), 71.0 (O*C*H_2_Ph), 66.4 (C_6_), 63.7 (C_2_), 57.5 (C_5_), 21.6 (CH_3_-Ts); **IR (cm^-1^)**: 3525 (w), 2905 (w), 2099 (vs); **HRMS (ESI) m/z =** [M+Na]^+^ calcd for C_20_H_22_N_4_O_5_SNa 453.1203; found 453.1186.

###### **1,6-Anhydro-4-*O*-benzyl-2-deoxy-2-fluoro-*N*-tosyl-β-D-glucopiperidinose 10d**

To a solution of epoxide **9** (30 mg, 0.08 mmol, 1.0 eq) in dry ethylene glycol (221 μL, 0.35 M) under Ar atm. at RT was added KHF_2_ (36 mg, 0.46 mmol, 6.0 eq). The reaction mixture was stirred at 210°C for 2 h. The reaction mixture was cooled down to RT, diluted with 5 % K_2_CO_3_ (5 mL) and the aqueous layer was extracted with CHCl_3_ (4x15 mL). The combined organic layers were dried over MgSO_4_ and concentrated under reduced pressure. The residue was purified over a chromatography column (PE/EtOAc 80:20 to 70:30) to afford fluorinated derivative **10d** (9 mg, 29 %) as an oil; **R*_f_* =** 0.32 (PE/AcOEt 60:40); $\left[ \boldsymbol{\alpha} \right]_{\mathbf{D}}^{\mathbf{20}}$ **=** -64.0 (*c* 0.4, CHCl_3_); **^1^H NMR (400 MHz, CDCl_3_): δ** 7.82 (d, 2H, *J* = 8.2 Hz, H-Ar), 7.36 – 7.29 (m, 3H, H-Ar), 7.25 – 7.19 (m, 4H, H-Ar), 5.84 – 5.79 (m, 1H, H-1), 4.57 – 4.36 (m, 4H, H-2, H-5, OC*H_2_*Ph), 3.94 (d, 1H, *J* = 19.3 Hz, H-3), 3.80 (d, 1H, *J* = 7.8 Hz, H-6’), 3.47 (dd, 1H, *J* = 7.8, 5.8 Hz, H-6), 3.43 (br.s, 1H, H-4), 2.39 (s, 3H, CH_3_-Ts), 2.23 (br.s, 1H, C_3_-O*H*); **^13^C NMR (100 MHz, CDCl_3_): δ** 144.3, 137.2, 135.9 (C^IV^, C-Ar), 129.7, 128.5, 127.9, 127.9, 127.7 (CH-Ar), 90.1 (d, *J* = 187.8 Hz, C_2_), 86.3 (d, *J* = 28.1 Hz, C_1_), 79.4 (d, *J* = 5.7 Hz, C_4_), 71.3 (d, *J* = 26.9 Hz, C_3_), 71.0 (O*C*H_2_Ph), 66.6 (C_6_), 57.6 (C_5_), 21.7 (CH_3_-Ts); **^19^F NMR (375 MHz, CDCl_3_): δ** -182.6; **HRMS (ESI) m/z =** [M+H]^+^ calcd for C_20_H_23_O_5_NFS 408.1275; found 408.1278.

###### **2-*C*-Allyl-1,6-anhydro-4-*O*-benzyl-2-deoxy-*N*-tosyl-β-D-glucopiperidinose 10e**

Compound **9** (30 mg, 0.08 mmol, 1.0 eq.) was treated with allylMgBr (1M/Et_2_O, 774 μL, 0.77 mmol, 10.0 eq.) and CuI (74 mg, 0.39 mmol, 5.0 eq.) following general **procedure C** to give allyl derivative **10e** (19 mg, 61 %) as a colorless oil. **R*_f_* =** 0.38 (PE/AcOEt 60:40); $\left[ \boldsymbol{\alpha} \right]_{\mathbf{D}}^{\mathbf{20}}$ **=** -8.0 (*c* 0.3, CHCl_3_); **^1^H NMR (400 MHz, CDCl_3_): δ** 7.78 (d, 2H, *J* = 8.2 Hz, H-Ar), 7.37 – 7.29 (m, 3H, H-Ar), 7.26 – 7.21 (m, 4H, H-Ar), 5.77 (ddt, 1H, *J* = 17.1, 10.2, 7.0 Hz, H-b), 5.69 (d, 1H, *J* = 2.5 Hz, H-1), 5.09 – 5.04 (m, 1H, H-c’), 5.00 (dd, 1H, *J* = 17.1, 1.6 Hz, H-c), 4.58 (d, 1H, *J* = 11.7 Hz, OCH*H*Ph), 4.49 (d, 1H, *J* = 11.7 Hz, OCH*H*Ph), 4.42 (dd, 1H, *J* = 4.7, 2.4 Hz, H-5), 3.94 (d, 1H, *J* = 7.9 Hz, H-6’), 3.69 (d, 1H, *J* = 5.4 Hz, H-3), 3.53 (br.s, 1H, H-4), 3.27 (dd, 1H, *J* = 7.9, 5.4 Hz, H-6), 2.40 (s, 3H, CH_3_-Ts), 2.33 – 2.11 (m, 3H, H-a’, H-a, C_3_-O*H*), 1.99 (t, 1H, *J* = 7.4 Hz, H-2); **^13^C NMR (100 MHz, CDCl_3_): δ** 144.4, 137.8, 136.1 (C^IV^, C-Ar), 135.5 (C_b_), 129.8, 128.5, 127.9, 127.8, 127.5 (CH-Ar), 117.7 (C_c_), 90.0 (C_1_), 80.3 (C_4_), 71.0 (O*C*H_2_Ph), 70.7 (C_3_), 65.7 (C_6_), 57.9 (C_5_), 47.9 (C_2_), 34.1 (C_a_), 21.8 (CH_3_-Ts); **HRMS (ESI) m/z =** [M+H]^+^ calcd for C_23_H_28_O_5_NS 430.1683; found 430,1680.

###### **1,6-Anhydro-3-azido-3-deoxy-*N*-tosyl-β-D-glucopiperidinose 10f**

To a solution of the mixture of epoxides **5** and **6** (20 mg, 0.07 mmol, 1.0 eq) in dry DMF (675 μL) under Ar atm. at RT was added NaN_3_ (18 mg, 0.27 mmol, 4.0 eq). The reaction mixture was stirred overnight at 90°C, cooled down to RT and concentrated under reduced pressure. The residue was diluted in Et_2_O (5 mL), washed with H_2_O (10 mL) and the aqueous phase was extracted with Et_2_O (3x10 mL). The combined organic layers were dried over MgSO_4_ and concentrated under reduced pressure to give azide **10f** (25 mg, quant.) as a white solid. **R*_f_* =** 0.80 (PE/AcOEt 20:80); mp: 157.0°C; $\left[ \boldsymbol{\alpha} \right]_{\mathbf{D}}^{\mathbf{20}}$ **=** -26.0 (*c* 0.1, CHCl_3_); **^1^H NMR (500 MHz, CD_3_OD): δ** 7.81 (d, 2H, *J* = 8.3 Hz, H-Ar), 7.40 (d, 2H, *J* = 8.0 Hz, H-Ar), 5.46 (s, 1H, H-1), 4.19 (d, 1H, *J* = 5.6 Hz, H-5), 3.52 (dd, 1H, *J* = 8.3, 0.7 Hz, H-2), 3.46 (m, 2H, H-6’, H-3), 3.36 (t, 1H, *J* = 5.1 Hz, H-4), 2.87 (dd, 1H, *J* = 8.2, 5.7 Hz, H-6), 2.44 (s, 3H, CH_3_-Ts); **^13^C NMR (125 MHz, CD_3_OD): δ** 146.2, 136.9 (C^IV^, C-Ar), 130.9, 129.1 (CH-Ar), 92.1 (C_1_), 74.6 (C_4_), 74.2 (C_2_), 68.0 (C_6_), 67.5 (C_3_), 63.1 (C_5_), 21.5 (CH_3_-Ts); **IR (cm^-1^)**: 3512 (w), 2922 (s), 2852 (s), 2098 (s); **HRMS (ESI) m/z =** [M+H]^+^ calcd for C_13_H_17_O_5_N_4_S 341.0914; found 341.0901.

###### **1,6-Anhydro-3-deoxy-3-*C*-methyl-*N*-tosyl-β-D-glucopiperidinose 10g**

A mixture of epoxides **5** and **6** (30 mg, 0.10 mmol, 1.0 eq.) was treated with methyl magnesium bromide (3M/Et_2_O, 336 μL, 1.01 mmol, 10.0 eq.) and CuI (11.5 mg, 0.06 mmol, 0.6 eq.) following general **procedure C** to give diol **10g** (12 mg, 39 %) as a white solid. **R*_f_* =** 0.24 (PE/AcOEt 40:60);$\left[ \boldsymbol{\alpha} \right]_{\mathbf{D}}^{\mathbf{20}}$ **=** -14.5 (*c* 0.4, MeOH); **^1^H NMR (500 MHz, CDCl_3_): δ** 7.75 (d, 2H, *J* = 8.2 Hz, H-Ar), 7.27 (d, 2H, *J* = 8.2 Hz, H-Ar), 5.57 (d, 1H, *J* = 3.2 Hz, H-1), 4.30 – 4.22 (m, 1H, H-5), 3.52 – 3.46 (m, 3H, H-2, H-4, H-6’), 2.85 (dd, 1H, *J* = 8.5, 5.5 Hz, H-6), 2.37 (s, 3H, CH_3_-Ts), 1.91 (q, 1H, *J* = 8.1 Hz, H-3), 1.09 (d, 3H, *J* = 8.1 Hz, CH_3_); **^13^C NMR (125 MHz, CDCl_3_): δ** 144.9, 135.4 (C^IV^, C-Ar), 130.2, 127.9 (CH-Ar), 90.5 (C_1_), 73.5 (C_2_ or C_4_), 72.4 (C_2_ or C_4_), 65.3 (C_6_), 61.9 (C_5_), 42.0 (C_3_), 21.8 (CH_3_-Ts), 19.8 (CH_3_); **HRMS (ESI) m/z =** [M+H]^+^ calcd for C_14_H_20_O_5_NS 314.1057; found 314.1044; **X-ray crystallography**: CCDC 2447466 (see p 36).

###### **3-*C*-allyl-1,6-anhydro-3-deoxy-*N*-tosyl-β-D-glucopiperidinose 10h**

A mixture of epoxides **5** and **6** (30 mg, 0.10 mmol, 1.0 eq.) were treated with allyl magnesium bromide (1M/Et_2_O, 1.01 mL, 1.01 mmol, 10.0 eq.) and CuI (11.5 mg, 0.06 mmol, 0.6 eq.) following general **procedure C** to give diol **10h** (17 mg, 50 %) as a white solid. **R*_f_* =** 0.51 (PE/AcOEt 40:60); $\left[ \boldsymbol{\alpha} \right]_{\mathbf{D}}^{\mathbf{20}}$ **=** -8.5 (*c* 0.4, MeOH); **^1^H NMR (400 MHz, CDCl_3_): δ** 7.81 (d, 2H, *J* = 8.2 Hz, H-Ar), 7.33 (d, 2H, *J* = 8.2 Hz, H-Ar), 5.75 (ddt, 1H, *J* = 17.1, 10.2, 6.9 Hz, H-b), 5.64 (d, 1H, *J* = 3.3 Hz, H-1), 5.11 – 5.00 (m, 2H, H-c, H-c’), 4.33 – 4.27 (m, 1H, H-5), 3.66 (s, 2H, H-2, H-4), 3.51 (d, 1H, *J* = 8.6 Hz, H-6’), 2.95 (dd, 1H, *J* = 8.6, 5.4 Hz, H-6), 2.74 (br.s, 1H, C_2_-O*H* or C_4_-O*H*), 2.51 (s, 1H, C_2_-O*H* or C_4_-O*H*), 2.44 (s, 3H, CH_3_-Ts), 2.29 – 2.09 (m, 2H, H-a, H-a’), 1.91 (t, 1H, *J* = 8.3 Hz, H-3); **^13^C NMR (100 MHz, CDCl_3_): δ** 145.0 (C^IV^, C-Ar), 136.4 (C_b_), 135.4 (C^IV^, C-Ar), 130.2, 127.9 (CH-Ar), 117.7 (C_c_), 90.3 (C_1_), 71.2 (C_2_ or C_4_), 70.3 (C_2_ or C_4_), 65.2 (C_6_), 61.8 (C_5_), 46.7 (C_3_), 38.1 (C_a_), 21.8 (CH_3_-Ts); **HRMS (ESI) m/z =** [M+H]^+^ calcd for C_16_H_22_O_5_NS 340.1213; found 340.1204; **X-ray crystallography**: CCDC 2447465 (see p 37).

###### **1,6-Anhydro-3-bromo-3-deoxy-*N*-tosyl-β-D-glucopiperidinose 10i**

To a solution of the mixture of epoxides **5** and **6** (30 mg, 0.10 mmol, 1.0 eq.) in dry Et_2_O (1 mL, 0.1 M) under Ar atm. at 0°C was added vinylMgBr (1M/THF, 504 μL, 0.50 mmol, 5.0 eq). After stirring overnight at RT, TLC showed total consumption of the starting material. The reaction mixture was quenched at 0°C with a saturated solution of NH_4_Cl (3 mL), then the resulting solution was extracted thrice with DCM (3x10 mL). The combined organic layers were dried over MgSO_4_, filtered and concentrated. The crude was purified over a flash chromatography column (PE/EtOAc 80:20 to 70:30) to give diol **10i** (18 mg, 47 %) as a white solid. **R*_f_* =** 0.76 (PE/AcOEt 40:60); $\left[ \boldsymbol{\alpha} \right]_{\mathbf{D}}^{\mathbf{20}}$ **=** -5.6 (*c* 0.5, MeOH); **^1^H NMR (400 MHz, CD_3_OD): δ** 7.82 (d, 2H, *J* = 8.2 Hz, H-Ar), 7.40 (d, 2H, *J* = 8.2 Hz, H-Ar), 5.50 (s, 1H, H-1), 4.26 (d, 1H, *J* = 5.6 Hz, H-5), 3.91 (dd, 1H, *J* = 4.4, 1.4 Hz, H-4), 3.86 (dd, 1H, *J* = 4.4, 1.4 Hz, H-2), 3.77 (d, 1H, *J* = 8.4 Hz, H-6’), 3.67 (t, 1H, *J* = 4.4 Hz, H-3), 2.91 (dd, 1H, *J* = 8.4, 5.6 Hz, H-6), 2.44 (s, 3H, CH_3_); **^13^C NMR (100 MHz, CD_3_OD): δ** 146.2, 136.9 (C^IV^, C-Ar), 131.0, 129.1 (CH-Ar), 92.1 (C_1_), 77.0 (C_2_), 76.9 (C_4_), 67.6 (C_6_), 63.4 (C_5_), 53.8 (C_3_), 21.6 (CH_3_-Ts); **IR (cm^-1^)**: 2913 (w), 2850 (w), 2107 (s), 1352 (s); **HRMS (ESI) m/z =** [M+H]^+^ calcd for C_13_H_17_O_5_NBrS 378.0005; found 377.9995; **X-ray crystallography**: CCDC 2447465 (see p 39).

###### **1,6:2,3-Dianhydro-4-azido-4-deoxy-*N*-tosyl-β-D-gulopiperidinose 10j**

Compound **5** (30 mg, 0.10 mmol, 1.0 eq.) was activated with Tf_2_O (38 μL, 0.23 mmol, 2.3 eq.) following general **procedure B** to give the expected triflate (40 mg, quant.) as a yellow oil, which was directly engaged in the following step without purification. To a solution of this triflate in dry DMF (1.00 mL, 0.1M) under Ar atm. at RT was added NaN_3_ (40 mg, 0.60 mmol, 6.0 eq). The reaction mixture was stirred at RT for 32 h, when TLC showed total consumption of the triflate. The reaction mixture was concentrated under reduced pressure. The residue was purified over a flash chromatography column (RediSep® 4 g, PE/EtOAc 90:10 to 50:50) to afford azide **10j** (18 mg, 55 %) as a colorless oil. **R*_f_* =** 0.61 (PE/AcOEt 70:30); $\left[ \boldsymbol{\alpha} \right]_{\mathbf{D}}^{\mathbf{20}}$ **=** +198.0 (*c* 0.1, CHCl_3_); **^1^H NMR (400 MHz, CDCl_3_): δ** 7.76 (d, 2H, *J* = 8.2 Hz, H-Ar), 7.32 (d, 2H, *J* = 8.2 Hz, H-Ar), 5.92 (d, 1H, *J* = 2.2 Hz, H-1), 4.23 – 4.12 (m, 2H, H-5, H-4), 3.79 (dd, 1H, *J* = 8.6, 1.2 Hz, H-6’), 3.24 – 3.19 (m, 1H, H-2), 3.10 (dd, 1H, *J* = 8.6, 6.5 Hz, H-6), 3.02 (dd, 1H, *J* = 3.6, 2.0 Hz, H-3), 2.44 (s, 3H, CH_3_-Ts); **^13^C NMR (100 MHz, CDCl_3_): δ** 144.0, 135.3 (C^IV^, C-Ar), 130.1, 127.9 (CH-Ar), 85.5 (C_1_), 64.3 (C_6_), 57.6 (C_4_), 56.4 (C_5_), 50.9 (C_3_), 50.4 (C_2_), 21.8 (CH_3_-Ts); **HRMS (ESI) m/z =** [M+H]^+^ calcd for C_13_H_15_O_4_N_4_S 323.0808; found 323.0809.

###### **1,6:2,3-Dianhydro-4-deoxy-4-fluoro-*N*-tosyl-β-D-gulopiperidinose 10k**

Alcohol **5** (37 mg, 0.12 mmol, 1.0 eq.) was activated with Tf_2_O (47 μL, 0.29, 2.3 eq.) following **procedure B** to give the expected triflate (55 mg, quant.) as a yellow oil, which was directly engaged in the following step without purification. To a solution of this triflate in dry DCM (1.24 mL, 0.1M) under Ar atm. at RT was added TBAF.3H_2_O (59 mg, 0.19 mmol, 3.0 eq). The reaction mixture was stirred at RT for 48 h, when TLC showed total consumption of the triflate. The reaction mixture was diluted with H_2_O (5 mL) and the aqueous layer was extracted with DCM (4x10 mL). The combined organic layers were dried over MgSO_4_ and concentrated under reduced pressure. The residue was purified over a flash chromatography column (RediSep® 4 g, PE/EtOAc 99:1 to 50:50) to afford fluorinated derivative **10k** (24 mg, 64 %) as a white solid. **R*_f_* =** 0.40 (PE/AcOEt 80:20); $\left[ \boldsymbol{\alpha} \right]_{\mathbf{D}}^{\mathbf{20}}$ **=** +19.0 (*c* 0.1, CHCl_3_); **^1^H NMR (400 MHz, CDCl_3_): δ** 7.76 (d, 2H, *J* = 8.2 Hz, H-Ar), 7.32 (d, 2H, *J* = 8.2 Hz, H-Ar), 5.93 (s, 1H, H-1), 4.93 (dd, 1H, *J* = 46.9, 5.1 Hz, H-4), 4.36 - 4.29 (m, 1H, H-5), 3.88 (d,1H, *J* = 8.9 Hz, H-6’), 3.28 - 3.24 (m, 1H, H-2), 3.19 - 3.09 (m, 2H, H-3, H-6), 2.44 (s, 3H, CH_3_-Ts); **^13^C NMR (100 MHz, CDCl_3_): δ** 145.0, 135.2 (C^IV^, C-Ar), 130.1, 127.9 (CH-Ar), 85.6 (C_1_), 83.5 (d, *J* = 181.5 Hz, C_4_), 63.7 (d, *J* = 1.3 Hz, C_6_), 55.6 (d, *J* = 28.4 Hz, C_5_), 50.3 (C_2_), 50.1 (d, *J* = 39.1 Hz, C_3_), 21.8 (CH_3_-Ts); **^19^F NMR (376 MHz, CDCl_3_): δ** -194.1 (s); **HRMS (ESI) m/z =** [M+H]^+^ calcd for C_13_H_15_O_4_NFS 300.0700; found 300.0693; **X-ray crystallography**: CCDC 2447467 (see p 41).

###### **1,6-anhydro-4-*C*-allyl-4-deoxy-2-*O*-tosyl-*N*-tosyl-β-D-glucopiperidinose 10l**

Epoxide **8** (50 mg, 0.11 mmol, 1.0 eq.) was treated with allyl magnesium bromide (1M/Et_2_O, 1.11 mL, 1.11 mmol, 10.0 eq.) and CuI (105 mg, 0.55 mmol, 5.0 eq.) following general **procedure C** to give alcohol **10l** (33 mg, 60 %) as a colorless oil. **R*_f_* =** 0.40 (PE/AcOEt 60:40); $\left[ \boldsymbol{\alpha} \right]_{\mathbf{D}}^{\mathbf{20}}$ **=** -30.8 (*c* 0.5, CHCl_3_); **^1^H NMR (400 MHz, CDCl_3_): δ** 7.79 (d, 2H, *J* = 8.2 Hz, H-Ar), 7.73 (d, 2H, *J* = 8.2 Hz, H-Ar), 7.38 (d, 2H, *J* = 8.2 Hz, H-Ar), 7.31 (d, 2H, *J* = 8.2 Hz, H-Ar), 5.64 (ddt, 1H, *J* = 17.1, 10.2, 7.1 Hz, H-b), 5.49 (d, 1H, *J* = 2.7 Hz, H-1), 5.04 (d, 1H, *J* = 10.2 Hz, H-c’), 4.92 (dd, 1H, *J* = 17.1, 1.5 Hz, H-c), 4.35 (t, 1H, *J* = 2.1 Hz, H-2), 4.13 (d, 1H, *J* = 4.0 Hz, H-5), 3.86 (d, 1H, *J* = 7.6 Hz, H-6’), 3.72 – 3.65 (m, 1H, H-3), 3.25 (dd, 1H, *J* = 7.6, 5.3 Hz, H-6), 2.48 (s, 3H, CH_3_-Ts), 2.44 (s, 3H, CH_3_-Ts), 2.32 (d, 1H, *J* = 5.8 Hz, C_3_-O*H*), 2.22 (dt, 1H, *J* = 13.7, 6.8 Hz, H-a’), 2.17 – 2.07 (m, 1H, H-a), 1.78 (t, 1H, *J* = 7.6 Hz, H-4); **^13^C NMR (100 MHz, CDCl_3_): δ** 145.7, 144.8, 135.6 (C^IV^, C-Ar), 135.1 (C_b_), 132.8 (C^IV^, C-Ar), 130.3, 130.0, 128.1, 128.1 (CH-Ar), 118.5 (C_c_), 86.9 (C_1_), 79.3 (C_2_), 71.2 (C_3_), 69.3 (C_6_), 57.5 (C_5_), 45.8 (C_4_), 35.7 (C_a_), 21.9 (CH_3_-Ts), 21.8 (CH_3_-Ts); **HRMS (ESI) m/z =** [M+H]^+^ calcd for C_23_H_28_O_7_NS_2_ 494.1302; found 494.1284.

###### **1,6-Anhydro-2,4-diazido-2,4-dideoxy-*N*-tosyl-β-D-glucopiperidinose 10m**

Condition A: To a solution of tosylate **7** (55 mg, 0.09 mmol, 1.0 eq) in dry DMF (882 μL) under Ar atm. at RT was added NaN_3_ (46 mg, 0.71 mmol, 8.0 eq). The reaction mixture was stirred for 30 min at 150W and 150°C using a Discover® 2.0 Microwave Reactor. Then, the reaction mixture was concentrated under reduced pressure and the residue was purified over a flash chromatography column (PE/EtOAc 85:15 to 60:40) to afford azide **10m** (15 mg, 48%) as a colorless oil.

Condition B: To a solution of epoxide **8** (30 mg, 0.07 mmol, 1.0 eq) in dry DMF (664 μL) under Ar atm. at RT was added NaN_3_ (26 mg, 0.42 mmol, 6.0 eq). The reaction mixture was stirred for 3 h at 120°C, when TLC showed total consumption of the starting material. The reaction mixture was concentrated under reduced pressure. The residue was purified over a flash chromatography column (PE/EtOAc 85:15 to 60:40) to afford azide **10m** (8 mg, 31%) as a colorless oil. **R*_f_* =** 0.72 (PE/AcOEt 50:50); $\left[ \boldsymbol{\alpha} \right]_{\mathbf{D}}^{\mathbf{25}}$ **=** -207.0 (*c* 0.1, CHCl_3_); **^1^H NMR (500 MHz, CDCl_3_): δ** 7.83 (d, 2H, *J* = 8.3 Hz, H-Ar), 7.34 (d, 2H, *J* = 8.1 Hz, H-Ar), 5.72 (d, 1H, *J* = 2.2 Hz, H-1), 4.35 (d, 1H, *J* = 5.2 Hz, H-5), 3.94 (d, 1H, *J* = 8.2 Hz, H-6’), 3.77 (br.s, 1H, H-3), 3.58 - 3.53 (m, 2H, H-2, H-4), 3.40 (dd, 1H, *J* = 8.2, 5.4 Hz, H-6), 2.70 (br.s, 1H, C_3_-O*H*), 2.45 (s, 3H, CH_3_-Ts); **^13^C NMR (126 MHz, CDCl_3_): δ** 145.2, 135.1 (C^IV^, C-Ar), 130.1, 128.1 (CH-Ar), 87.9 (C_1_), 71.5 (C_3_), 67.4 (C_6_), 64.2 (C_2_ or C_4_), 64.2 (C_2_ or C_4_), 58.4 (C_5_), 21.8 (CH_3_-Ts); **IR (cm^-1^)**: 3532 (w), 2905 (w), 2099 (vs); **HRMS (ESI) m/z =** [M+H]^+^ calcd for C_13_H_16_O_4_N_7_S 366.0979; found 366.0966.

###### **1,6-Anhydro-2,4-dideoxy-2,4-difluoro-*N*-tosyl-β-D-glucopiperidinose 10n**

To a solution of tosylate **7** (50 mg, 0.08 mmol, 1.0 eq) in dry ethylene glycol (802 μL, 0.1 M) under Ar atm. at RT were added KHF_2_ (37 mg, 0.48 mmol, 6.0 eq) and KF (28 mg, 0.48 mmol, 6.0 eq). The reaction mixture was stirred at 170°C for 2 h. The reaction mixture was cooled down to RT, diluted with H_2_O (5 mL) and the aqueous layer was extracted with AcOEt (4x15 mL). The combined organic layers were dried over MgSO_4_ and concentrated under reduced pressure. The residue was purified over a chromatography column (PE/EtOAc 100:0 to 0:100) to afford fluorinated derivative **10n** (4 mg, 16 %) as a colorless oil. **R*_f_* =** 0.19 (PE/AcOEt 50:50); $\left[ \boldsymbol{\alpha} \right]_{\mathbf{D}}^{\mathbf{20}}$ **=** +125.0 (*c* 0.2, CHCl_3_); **^1^H NMR (400 MHz, acetone-*d*_6_): δ** 7.84 (d, 2H, *J* = 8.0 Hz, H-Ar), 7.44 (d, 2H, *J* = 8.0 Hz, H-Ar), 5.79 (br.s, 1H, H-1), 5.06 (d, 1H, *J* = 4.6 Hz, C_3_-O*H*), 4.67 – 4.52 (m, 2H, H-5, H-4), 4.45 (d, 1H, *J* = 46.0 Hz, H-2), 4.04 – 3.88 (m, 1H, H-3), 3.83 (d, 1H, *J* = 8.0 Hz, H-6’), 3.05 – 2.97 (m, 1H, H-6), 2.45 (s, 3H, CH_3_-Ts); **^13^C NMR (100 MHz, acetone-*d*_6_): δ** 144.5, 136.1 (C^IV^, C-Ar), 129.7, 127.6 (CH-Ar), 90.8 (dd, *J* = 184.1, 5.2 Hz, C_4_), 89.6 (dd, *J* = 184.4, 4.3 Hz, C_2_), 86.2 (d, *J* = 27.8 Hz, C_1_), 70.0 (t, *J* = 29.3 Hz, C_3_), 63.8 (d, *J* = 8.8 Hz, C_6_), 58.1 (d, *J* = 20.5 Hz, C_5_), 20.5 (CH_3_-Ts); **^19^F NMR (376 MHz, acetone-*d*_6_): δ *(ppm)* =** -178.0 (s), -183.2 (s); **HRMS (ESI) m/z =** [M+Na]^+^ calcd for C_13_H_15_O_4_NF_2_NaS 342.0588; found 342.0576.

###### **2,4-Di-*C*-allyl-1,6-anhydro-2,4-dideoxy-*N*-tosyl-β-D-glucopiperidinose 10o**

To a solution of tosylate **10l** (35 mg, 0.07 mmol, 1.0 eq) in dry MeOH (709 μL) under Ar atm. at 0°C was added Na° (16 mg, 0.70 mmol, 10.0 eq). The reaction was stirred at RT for 4 h, when TLC showed total consumption of the starting material. The reaction mixture was neutralized with addition of Amberlite IR 120H resin. After filtration and concentration under reduced pressure, the crude was taken up in DCM/H_2_O and the aqueous phase was extracted with DCM (3x10 mL). The combined organic layers were dried over MgSO_4_ and concentrated under reduced pressure to afford **4-*C*-allyl-1,6:2,3-dianhydro-4-deoxy-N-tosyl-β-D-mannopiperidinose** (22 mg, quant.) as a white foam. **R*_f_* =** 0.83 (PE/AcOEt 60:40); $\left[ \boldsymbol{\alpha} \right]_{\mathbf{D}}^{\mathbf{20}}$ **=** +73.8 (*c* 0.5, CHCl_3_); **^1^H NMR (400 MHz, CDCl_3_): δ** 7.73 (d, 2H, *J* = 8.2 Hz, H-Ar), 7.29 (d, 2H, *J* = 8.2 Hz, H-Ar), 5.90 (d, 1H, *J* = 4.3 Hz, H-1), 5.79 (ddt, 1H, *J* = 17.3, 10.1, 7.1 Hz, H-b), 5.25 – 5.11 (m, 2H, H-c), 3.90 (d, 1H, *J* = 6.3 Hz, H-5), 3.54 (t, 1H, *J* = 4.0 Hz, H-2), 3.43 (d, 1H, *J* = 7.1 Hz, H-6’), 2.88 (d, 1H, *J* = 3.6 Hz, H-3), 2.76 (t, 1H, *J* = 7.1 Hz, H-6), 2.42 (s, 3H, CH_3_-Ts), 2.37 – 2.23 (m, 2H, H-a’, H-a), 2.12 – 2.05 (m, 1H, H-4); **^13^C NMR (100 MHz, CDCl_3_): δ** 144.7, 135.0 (C^IV^, C-Ar), 134.8 (C_b_), 129.9, 128.2 (CH-Ar), 118.9 (C_c_), 85.4 (C_1_), 68.6 (C_6_), 55.3 (C_2_), 54.8 (C_5_), 51.6 (C_3_), 41.2 (C_4_), 34.6 (C_a_), 21.8 (CH_3-_Ts); **HRMS (ESI) m/z =** [M+H]^+^ calcd for C_16_H_20_O_4_NS 322.1108; found 322.1115.

The **4-*C*-allyl-1,6:2,3-dianhydro-4-deoxy-N-tosyl-β-D-mannopiperidinose** (20 mg, 0.06 mmol, 1.0 eq.) was treated with allyl magnesium bromide (1M/Et_2_O, 622 μL, 0.62mmol, 10.0 eq.) and CuI (59.3 mg, 0.31 mmol, 5.0 eq.) following general **procedure C** to give diallyl derivative **10o** (9 mg, 41 %, 2 steps) as a colorless oil. **R*_f_* =** 0.42 (PE/AcOEt 60:40); $\left[ \boldsymbol{\alpha} \right]_{\mathbf{D}}^{\mathbf{20}}$ **=** -13.6 (*c* 0.5, CHCl_3_); **^1^H NMR (500 MHz, CDCl_3_): δ** 7.75 (d, 2H, *J* = 8.2 Hz, H-Ar), 7.31 (d, 2H, *J* = 8.2 Hz, H-Ar), 5.86 – 5.71 (m, 2H, H-b, H-e), 5.61 (d, 1H, *J* = 2.4 Hz, H-1), 5.11 (m, 4H, H-c’, H-c, H-f’, H-f), 4.13 – 4.08 (m, 1H, H-5), 3.88 (d, 1H, *J* = 7.4 Hz, H-6’), 3.49 (s, 1H, H-3), 2.94 (dd, 1H, *J* = 7.4, 5.3 Hz, H-6), 2.44 (s, 3H, CH_3_-Ts), 2.35 – 2.19 (m, 3H, H-d’, H-d, H-a’), 2.17 – 2.10 (m, 1H, H-a), 1.98 (t, 1H, *J* = 7.5 Hz, H-2), 1.88 – 1.83 (m, 1H, H-4); **^13^C NMR (125 MHz, CDCl_3_): δ** 144.6 (C^IV^, C-Ar), 135.8 (C_b_ or C_e_), 135.7 (C^IV^, C-Ar), 135.4 (C_b_ or C_e_), 130.0, 127.9 (CH-Ar), 118.1 (C_c_ or C_f_), 117.9 (C_c_ or C_f_), 90.3 (C_1_), 72.0 (C_3_), 68.4 (C_6_), 58.0 (C_5_), 49.5 (C_2_), 48.0 (C_4_), 36.5 (C_d_), 35.1 (C_a_), 21.8 (CH_3_-Ts); **HRMS (ESI) m/z =** [M+H]^+^ calcd for C_19_H_26_O_4_NS 364.1577; found 364.1572.

###### **2,3,4-Tri-*O*-benzyl-*N*-tosyl-1-deoxynojirimycin 11a**

Hemiaminal **3** (50 mg, 0.08 mmol, 1.0 eq.) was reacted with Et_3_SiH (54 μL, 0.32 mmol, 4.0 eq.) and TiCl_4_ (1M/DCM, 43 μL, 0.04 mmol, 0.5 eq.) following general **procedure D** to give piperidine **11a** (33 mg, 66 %) as a colorless oil. **R*_f_* =** 0.22 (PE/AcOEt 80:20); $\left[ \boldsymbol{\alpha} \right]_{\mathbf{D}}^{\mathbf{20}}$ **=** +10.6 (*c* 0.5, CHCl_3_); **^1^H NMR (400 MHz, CDCl_3_): δ** 7.66 (d, 2H, *J* = 8.2 Hz, H-Ar), 7.30 – 7.16 (m, 15H, H-Ar), 7.05 – 6.99 (m, 2H, H-Ar), 4.58 – 4.45 (m, 4H, 2xOC*H_2_*Ph), 4.36 (d, 1H, *J* = 11.5 Hz, OCH*H*Ph), 4.25 (d, 1H, *J* = 11.5 Hz, OCH*H*Ph), 3.91 – 3.75 (m, 3H, H-6’, H-6, H-1’), 3.74 – 3.66 (m, 1H, H-2), 3.64 (t, 1H, *J* = 4.4 Hz, H-4), 3.55 – 3.49 (m, 1H, H-5, H-3), 3.22 (dd, 1H, *J* = 12.2, 7.7 Hz, H-1), 2.66 (br.s, 1H, C_6_-O*H*), 2.24 (s, 3H, CH_3_-Ts); **^13^C NMR (100 MHz, CDCl_3_): δ** 143.6, 137.8, 137.7, 137.7, 135.13 (C^IV^, C-Ar), 129.5, 128.6, 128.5, 128.4, 128.1, 127.9, 127.9, 127.8, 127.7 (CH-Ar), 80.1 (C_3_), 77.1 (C_2_), 75.9 (C_4_), 73.3, 72.5, 71.9 (O*C*H_2_Ph), 62.0 (C_6_), 59.7 (C_5_), 43.9 (C_1_), 21.6 (CH_3_-Ts); **HRMS (ESI) m/z =** [M+H]^+^ calcd for C_34_H_38_O_6_NS 588.2414; found 588.2399.

###### **2,4-Di-*O*-tosyl-*N*-tosyl-1-deoxynojirimycin 11b**

Hemiaminal **7** (50 mg, 0.08 mmol, 1.0 eq.) was treated with Et_3_SiH (51 μL, 0.32 mmol, 4.0 eq.) and TiCl_4_ (1M/DCM, 40 μL, 0.04 mmol, 0.5 eq.) following general **procedure D** to give piperidine **11b** (29 mg, 58 %) as a colorless oil. **R*_f_* =** 0.37 (PE/AcOEt 50:50); $\left[ \boldsymbol{\alpha} \right]_{\mathbf{D}}^{\mathbf{20}}$ **=** -10.7 (*c* 0.6, CHCl_3_); **^1^H NMR (400 MHz, CDCl_3_): δ** 7.71 – 7.56 (m, 6H, H-Ar), 7.38 – 7.28 (m, 4H, H-Ar), 7.09 (m, 2H, H-Ar), 4.67 – 4.61 (m, 1H, H-5), 4.42 – 4.34 (m, 1H, H-2), 4.20 (d, 1H, *J* = 6.3 Hz, C_3_-O*H*), 4.03 – 3.92 (m, 2H, H-6’ and H-4), 3.89 – 3.80 (m, 2H, H-6 and H-3), 3.76 – 3.60 (m, 2H, H-1’ and H-1), 3.09 (s, 1H, C_6_-O*H*), 2.46 (s, 6H, 3xCH_3_), 2.36 (s, 3H, CH_3_); **^13^C NMR (100 MHz, CDCl_3_): δ** 145.5, 145.4, 143.9, 135.6, 132.9, 132.9 (C^IV^, C-Ar), 130.1, 130.1, 129.6, 128.0, 128.0, 127.9 (CH-Ar), 75.8 (C_5_), 74.7 (C_2_), 66.6 (C_3_), 63.2 (C_6_), 57.7 (C_4_), 42.1 (C_1_), 21.9, 21.9, 21.8 (CH_3_-Ts); **HRMS (ESI) m/z =** [M+H]^+^ calcd for C_27_H_32_O_10_NS_3_ 626.1183; found 626.1181.

###### **3-Azido-2,4-di-O-benzyl-*N*-tosyl-1,3-dideoxynojirimycin 11c**

To a stirred solution of hemiaminal **10f** (20 mg, 0.06 mmol, 1.0 eq.) in dry DMF (1 mL), at 0°C under Ar, were added BnBr (21 μL, 0.18 mmol, 3.0 eq.), then NaH (6.0 mg, 0.18 mmol, 3.0 eq.). The mixture was stirred at RT for 4h, quenched by addition of water (5 mL) and extracted twice with Et_2_0 (2 x 10 mL). The combined organic layers were dried over MgSO_4_ and concentrated to give the crude di-benzylated compound as a colorless oil. This crude (31 mg) was directly reacted with Et_3_SiH (37 μL, 0.23 mmol, 4.0 eq.) and TiCl_4_ (1M/DCM, 30 μL, 0.03 mmol, 0.5 eq.) following general **procedure D.** After 1h30, 3h and 4h30, Et_3_SiH (37 μL, 0.44 mmol, 4.0 eq.) and TiCl_4_ (1M/DCM, 30 μL, 0.03 mmol, 0.5 eq.) were added again and the mixture was stirred for 2 h at -50°C to give after purification azide **11c** (17.5 mg, 57 % over 2 steps) as a colorless oil. **R*_f_* =** 0.25 (PE/AcOEt 80:20); $\left[ \boldsymbol{\alpha} \right]_{\mathbf{D}}^{\mathbf{20}}$ **=** +19.5 (*c* 1.0, CHCl_3_); **^1^H NMR (400 MHz, CDCl_3_): δ** 7.57 (d, *J* = 8.3 Hz, 2H, H-Ar), 7.35 – 7.15 (m, 12H, H-Ar), 4.73 (d, *J* = 10.6 Hz, 1H, OCHPh), 4.61 (s, 2H, OCH_2_Ph), 4.56 (d, *J* = 10.6 Hz, 1H, OCHPh), 4.07 – 3.95 (m, 2H, H-1’, H-6’), 3.79 (m, 1H, H-6), 3.55 (t, *J* = 8.6 Hz, 1H, H-4), 3.45 (m, 1H, H-2), 3.27 (t, *J* = 8.8 Hz, 1H, H-3), 2.72 (m, 3H, OH, H-1, H-5), 2.32 (s, 3H, CH_3_); **^13^C NMR (100 MHz, CDCl_3_): δ** 144.3, 137.2, 137.1, 134.4 (C^IV^, C-Ar), 129.9, 128.6, 128.5, 128.1, 128.0, 127.3 (CH-Ar), 76.3 (C_4_), 76.1 (C_2_), 72.6 and 75.0 (OCH_2_Ph), 68.9 (C_3_), 63.1 (C_5_), 59.5 (C_6_), 48.7 (C_1_), 21.5 (CH_3_); **HRMS (ESI) m/z =** [M+Na]^+^ calcd for C_27_H_30_N_4_O_5_SNa 545.1829, found 545.1821.

###### **4-*C*-Allyl-4-deoxy-2-O-tosyl-*N*-tosyl-deoxynojirimycin 11d**

Hemiaminal **10l** (27 mg, 0.06 mmol, 1.0 eq.) was reacted with Et_3_SiH (71 μL, 0.44 mmol, 8.0 eq.) and TiCl_4_ (1M/DCM, 28 μL, 0.03 mmol, 0.5 eq.) following general **procedure D.** After 1h, Et_3_SiH (71 μL, 0.44 mmol, 8.0 eq.) and TiCl_4_ (1M/DCM, 28 μL, 0.03 mmol, 0.5 eq.) were added and the mixture was stirred 2 h at -50°C to give piperidine **11d** (12 mg, 44 %) as a colorless oil. **R*_f_* =** 0.16 (PE/AcOEt 60:40); $\left[ \boldsymbol{\alpha} \right]_{\mathbf{D}}^{\mathbf{20}}$ **=** +189.2 (*c* 0.6, CHCl_3_); **^1^H NMR (500 MHz, CDCl_3_): δ** 7.73 – 7.69 (m, 4H, H-Ar), 7.35 (d, 2H, *J* = 8.0 Hz, H-Ar), 7.27 (d, 2H, *J* = 8.0 Hz, H-Ar), 5.53 (ddt, 1H, *J* = 17.0, 10.2, 7.0 Hz, H-b), 5.00 (dd, 1H, *J* = 10.2, 1.5 Hz, H-c’), 4.81 (dd, 1H, *J* = 17.1, 1.5 Hz, H-c), 4.29 (dd, 1H, *J* = 5.7, 2.4 Hz, H-2), 3.97 (dd, 1H, *J* = 11.0, 3.2 Hz, H-6’), 3.81 (dd, 1H, *J* = 15.2, 1.9 Hz, H-1’), 3.77 – 3.67 (m, 3H, H-1, H-5 and H-6), 3.54 (t, 1H, *J* = 3.7 Hz, H-3), 2.46 (s, 3H, CH_3_), 2.43 (s, 3H, CH_3_), 2.05 – 1.93 (m, 2H, H-a and H-a’), 1.85 – 1.79 (m, 1H, H-4); **^13^C NMR (125 MHz, CD_3_OD+ 0.01% CDCl_3_): δ** 145.5, 144.0, 136.6 (C^IV^, C-Ar), 135.5 (CH, C_b_), 132.9 (C^IV^, C-Ar), 130.2, 129.8, 128.0, 127.9 (CH-Ar), 118.3 (C_c_), 77.5 (C_2_), 67.9 (C_3_), 65.7 (C_6_), 55.4 (C_5_), 42.7 (C_1_), 41.1 (C_4_), 35.4 (C_a_), 21.9 (CH_3_), 21.7 (CH_3_); **HRMS (ESI) m/z =** [M+Na]^+^ calcd for C_23_H_29_O_7_NNaS_2_ 518.1278; found 518.1263.

###### **2,3,4-Tri-*O*-benzyl-1-deoxynojirimycin 12a**

To a suspension of LiAlH_4_ (20 mg, 0.51 mmol, 6.0 eq.) in dry THF (0.8 mL) under Ar atm. at RT was added dropwise a solution of hemiaminal **3** (50 mg, 0.085 mmol, 1.0 eq) in dry THF (0.8 mL). The mixture was stirred at 85°C for 6h when TLC showed total consumption of the starting material. The reaction mixture was quenched at 0°C with 10 % NaOH, then stirred for 10 min, filtered and concentrated. The crude was purified over a flash chromatography column (RediSep® 4 g PE/EtOAc 30:70 to 0:100) to give piperidine **12a** (29 mg, 78 %) as a colorless oil. **R*f* =** 0.44 (PE/AcOEt 0:100); **^1^H NMR (400 MHz, CDCl_3_):** δ 7.40 – 7.27 (m, 15H, H-Ar), 5.00 (d, 1H, *J* = 10.9 Hz, OCH*H*Ph), 4.91 (d, 1H, *J* = 11.0 Hz, OCH*H*Ph), 4.86 (d, 1H, *J* = 10.9 Hz, OCH*H*Ph), 4.72 (d, 1H, *J* = 11.6 Hz, OCHHPh), 4.67 (d, 1H, *J* = 11.6 Hz, OCHHPh), 4.63 (d, 1H, *J* = 11.0 Hz, OCH*H*Ph), 3.76 (dd, 1H, *J* = 10.9, 2.9 Hz, H-6’), 3.63 - 3.55 (m, 2H, H-6, H-3), 3.54 – 3.46 (m, 1H, H-2), 3.33 (t, 1H, *J* = 9.3 Hz, H-4), 3.26 (dd, 1H, *J* = 12.0, 5.0 Hz, H-1’), 2.66 – 2.59 (m, 1H, H-5), 2.54 (dd, 1H, *J* = 12.0, 10.6 Hz, H-1). Analytical data are in agreement with the literature: M. Iftikhar, Z. Fang, *J. Carbohydr. Chem.* **2017**, 36, 295-306.

###### **2,4-di-*C*-Allyl-1,2,4-trideoxy-nojirimycin 12b**

Hemiaminal **10o** (15 mg, 0.041 mmol, 1.0 eq.) was reacted with LiAlH_4_ (9.4 mg, 0.247 mmol, 6.0 eq.) following general **procedure E** to give piperidine **12b** (4.4 mg, 51 %) as a colorless oil. **R*_f_* =** 0.25 (EtOAc/MeOH 60:40); $\left[ \boldsymbol{\alpha} \right]_{\mathbf{D}}^{\mathbf{20}}$ **=** +6.7 (*c* 0.43, CH_3_OH); **^1^H NMR (400 MHz, methanol-*d*_4_): δ** 5.92 – 5.74 (m, 2H, 2 x H-8), 5.18 – 5.02 (m, 4H, 4 x H-9), 3.85 (dd, *J* = 11.5, 3.0 Hz, 1H, H-6), 3.57 (dd, *J* = 11.5, 6.8 Hz, 1H, H-6’), 3.23 – 3.14 (m, 2H, H-1, H-3), 2.80 – 2.73 (m, 1H, H-5), 2.67 – 2.52 (m, 2H, 2 x H-7), 2.46 (t, *J* = 12.5 Hz, 1H, H-1’), 2.34 – 2.24 (m, 1H, H-7), 1.97 – 1.89 (m, 1H, H-7), 1.65 - 1.56 (m, 2H, H-2, H-4); **^13^C NMR (100 MHz, methanol-*d*_4_): δ** 136.8 (C_8_), 135.3 (C_8_), 118.3 (C_9_), 117.6 (C_9_), 72.8 (C_3_), 61.8 (C_6_), 60.9 (C_5_), 44.3(C_4_), 43.4 (C_2_), 35.2 (C_7_), 31.4 (C_7_); **HRMS (ESI) m/z =** [M+H]^+^ calcd for C_12_H_22_NO_2_ 212.1645; found 212.1641.

###### **2-*O*-benzyl-1,3-dideoxynojirimycin 12c**

To a stirred solution of compound **6** (20 mg, 0.067 mmol, 1.0 eq.) in dry DMF (1 mL), at 0°C under Ar, were added BnBr (16 μL, 0.134 mmol, 2.0 eq.), then NaH (4.8 mg, 0.134 mmol, 2.0 eq.). The mixture was stirred at RT for 3h, quenched by addition of water (5 mL) and extracted twice with Et_2_0 (2 x 10 mL). The combined organic layers were dried over MgSO_4_ and concentrated to give the crude benzylated compound as a colorless oil. This crude benzylated compound (25 mg) was reacted with LiAlH_4_ (15.2 mg, 0.40 mmol, 6.0 eq.) following general **procedure E** to give **12c** (5.8 mg, 36 % over 2 steps) as a colorless oil. **R*_f_* =** 0.25 (EtOAc/MeOH 80:20); $\left[ \boldsymbol{\alpha} \right]_{\mathbf{D}}^{\mathbf{20}}$ **=** +8.3 (*c* 0.42, CH_3_OH); **^1^H NMR (500 MHz, methanol-*d*_4_): δ** 7.46 - 7.10 (m, 5H, H-Ar), 4.55 (s, 2H, OC*H*_2_Ph), 3.81 (dd, 1H, *J* = 11.0, 3.2 Hz, H-6’), 3.53 (dd, 1H, *J* = 11.0, 6.6 Hz, H-6), 3.45 (td, 1H, *J* = 10.7, 5.2 Hz, H-2), 3.36 - 3.29 (m, 1H, H-4), 3.27 - 3.21 (m, 1H, H-1’), 2.43 - 2.29 (m, 3H, H-3’, H-5, H-4), 1.37 – 1.25 (m, 1H, H-3); **^13^C NMR (125 MHz, methanol-*d*_4_): δ** 139.9 (C^IV^, C-Ar), 129.4, 128.9, 128.7 (CH-Ar), 74.6 (C_2_), 71.7 (O*C*H_2_Ph), 67.8 (C_4_), 64.2 (C_5_), 62.8 (C_6_), 50.5 (C_1_), 41.3 (C_3_); **HRMS (ESI) m/z =** [M+H]^+^ calcd for C_13_H_20_NO_3_ 238.1438; found 238.1434.

###### **3-amino-2,4-di-*O*-benzyl-1,3-dideoxynojirimycin 12d**

To a stirred solution of compound **10f** (20 mg, 0.06 mmol, 1.0 eq.) in dry DMF (1 mL), at 0°C under Ar, were added BnBr (21 μL, 0.18 mmol, 3.0 eq.), then NaH (6.0 mg, 0.18 mmol, 3.0 eq.). The mixture was stirred at RT for 4h, quenched by addition of water (5 mL) and extracted twice with Et_2_0 (2 x 10 mL). The combined organic layers were dried over MgSO_4_ and concentrated to give the crude di-benzylated compound as a yellow oil, which was directly reacted with LiAlH_4_ (14 mg, 0.36 mmol, 6.0 eq.) following general **procedure E** to give **12d** (8.2 mg, 41 % over 2 steps) as a colorless oil. **R*_f_* =** 0.37 (EtOAc/MeOH 70:30); $\left[ \boldsymbol{\alpha} \right]_{\mathbf{D}}^{\mathbf{20}}$ **=** +24.8 (*c* 0.66, CH_3_OH); **^1^H NMR (500 MHz, methanol-*d*_4_): δ** 7.40 – 7.25 (m, 10H, H-Ar), 4.69 – 4.66 (m, 3H, OCH*H*Ph), 4.55 (d, 1H, OCH*H*Ph), 3.77 (dd, 1H, *J* = 10.9, 2.6 Hz, H-6’), 3.65 (dd, 1H, *J* = 10.9, 5.4 Hz, H-6), 3.31 (dd, 1H, *J* = 9.8, 3.2 Hz, H-1’), 3.26 (dd, 1H, *J* = 9.9, 4.7 Hz, H-2), 3.21 (t, 1H, *J* = 9.7 Hz, H-4), 2.84 (t, 1H, *J* = 9.5 Hz, H-3), 2.53 (ddd, 1H, *J* = 9.6, 5.4, 2.6 Hz, H-5), 2.39 (dd, 1H, *J* = 11.7; 10.0 Hz, H-1); **^13^C NMR (125 MHz, methanol-*d*_4_): δ** 139.8, 139.7 (C^IV^, C-Ar), 129.5, 129.1, 129.0, 128.9, 128.8 (CH-Ar), 81.4 (C_4_), 80.4 (C_2_), 75.6, 73.0 (O*C*H_2_Ph), 62.3 (C_5_), 62.2 (C_6_), 60.8 (C_3_), 48.2 (C_1_); **HRMS (ESI) m/z =** [M+H]^+^ calcd for C_20_H_27_N_2_O_3_ 343.2016; found 343.2013.

###### **α-1C-allyl-2,3,4-tri-*O*-benzyl-*N*-tosyl-1-deoxynojirimycin 13a-α and β-1C-allyl-2,3,4-tri-*O*-benzyl-*N*-tosyl-1-deoxynojirimycin 13a-β**

Compound **3** (50 mg, 0.08 mmol, 1.0 eq.) was reacted with TMS-allyl (110 μL, 0.68 mmol, 8.0 eq.) and TiCl_4_ (1M/DCM, 43 μL, 0.04 mmol, 0.5 eq.) following general **procedure D** to give compounds **13a-α** (19 mg, 36 %) and **13a-β** (25 mg, 47 %) as colorless oils.

**Data for compound 13a-α: R*_f_* =** 0.41 (PE/AcOEt 80:20); $\left[ \boldsymbol{\alpha} \right]_{\mathbf{D}}^{\mathbf{20}}$ **=** -15.0 (*c* 0.3, CHCl_3_); **^1^H NMR (500 MHz, acetone-*d*_6_): δ** 7.85 – 7.78 (m, 2H, H-Ar), 7.46 – 7.28 (m, 17H, H-Ar), 5.66 (dddd, 1H, *J* = 16.7, 10.2, 7.7, 6.5 Hz, H-b), 5.08 (dq, 1H, *J* = 17.1, 1.6 Hz, H-c’), 4.94 (ddt, 1H, *J* = 10.1, 2.1, 1.0 Hz, H-c), 4.87 (d, 1H, *J* = 11.3 Hz, OCH*H*Ph), 4.80 – 4.66 (m, 5H, OCH*H*Ph), 4.34 (dt, 1H, *J* = 9.6, 5.6 Hz, H-1), 4.15 – 4.01 (m, 2H, H-6’ and H-6), 3.80 (dd, 1H, *J* = 9.0, 7.1 Hz, H-3), 3.71 (dd, 1H, *J* = 9.0, 5.7 Hz, H-2), 3.66 (dd, 1H, *J* = 9.5, 7.2 Hz, H-4), 3.61 – 3.54 (m, 2H, H-5 and C_6_-O*H*), 2.67 – 2.58 (m, 1H, H-a’), 2.58 – 2.49 (m, 1H, H-a), 2.44 (s, 3H, CH_3_); **^13^C NMR (125 MHz, acetone-*d*_6_): δ** 144.3, 134.0, 139.9, 139.4 (C^IV^, C-Ar), 136.1 (C_b_), 130.4, 129.2, 129.1, 129.0, 128.8, 128.8, 128.6, 128.5, 128.4, 128.3, 128.2 (CH-Ar), 117.3 (C_c_), 83.4 (C_3_), 80.6 (C_2_), 79.5 (C_4_), 75.3, 74.9, 73.0 (O*C*H_2_Ph), 60.8 (C_6_), 60.6 (C_5_), 57.1 (C_1_), 31.3 (C_a_), 21.4 (CH_3_); **HRMS (ESI) m/z =** [M+H]^+^ calcd for C_37_H_42_O_6_NS 628.2727; found 628.2721.

**Data for compound 13a-β: R*_f_* =** 0.25 (PE/AcOEt 80:20); $\left[ \boldsymbol{\alpha} \right]_{\mathbf{D}}^{\mathbf{20}}$ **=** +6.0 (*c* 0.3, CHCl_3_); **^1^H NMR (500 MHz, acetone-*d*_6_): δ** 7.79 (d, 2H, *J* = 8.4 Hz, H-Ar), 7.35 – 7.27 (m, 11H, H-Ar), 7.27 – 7.23 (m, 4H, H-Ar), 7.07 (d, 2H, *J* = 8.4 Hz, H-Ar), 5.94 (dddd, 1H, *J* = 16.6, 10.3, 8.0, 6.3 Hz, H-b), 5.14 – 5.04 (m, 2H, H-c’ and H-c), 4.63 (d, 1H, *J* = 11.6 Hz, OCH*H*Ph), 4.58 – 4.53 (m, 4H, OCH*H*Ph), 4.48 (d, 1H, *J* = 11.3 Hz, OCH*H*Ph), 4.21 (dd, 1H, *J* = 6.4, 4.5 Hz, C_6_-O*H*), 4.06 (ddd, 1H, *J* = 9.3, 6.1, 3.4 Hz, H-1), 4.02 (ddd, 1H, *J* = 8.2, 5.0, 3.1 Hz, H-5), 3.97 – 3.90 (m, 2H, H-4 and H-6’), 3.85 – 3.80 (m, 1H, H-6), 3.66 (dd, 1H, *J* = 6.1, 3.4 Hz, H-2), 3.41 (t, 1H, *J* = 6.1 Hz, H-3), 2.69 – 2.56 (m, 2H, H-a’ and H-a), 2.27 (s, 3H, CH_3_); **^13^C NMR (125 MHz, acetone-*d*_6_): δ** 143.7, 139.5, 139.4, 139.4, 138.3 (C^IV^, C-Ar), 136.4 (C_b_), 129.9, 129.1, 128.9, 128.9, 128.8, 128.6, 128.5, 128.4, 128.3, 128.1, 128.1 (CH-Ar), 117.7 (C_c_), 79.6 (C_3_), 78.5 (C_2_), 76.3 (C_4_), 74.1, 72.6, 72.5 (O*C*H_2_Ph), 64.4 (C_6_), 60.4 (C_5_), 58.5 (C_1_), 41.7 (C_a_), 21.4 (CH_3_); **HRMS (ESI) m/z =** [M+H]^+^ calcd for C_37_H_42_O_6_NS 628.2727; found 628.2701.

**1α-azido-2,3,4-tri-*O*-benzyl-*N*-tosyl-1-deoxynojirimycin 13b-α and 1β-azido-2,3,4-tri-*O*-benzyl-*N*-tosyl-1-deoxynojirimycin 13b-β**

Compound **3** (50 mg, 0.08 mmol, 1.0 eq.) was treated with TMSN_3_ (45 μL, 0.34 mmol, 4.0 eq.) and TiCl_4_ (1M/DCM, 43 μL, 0.04 mmol, 0.5 eq.) following general **procedure D** to give compounds **13b-α** (19 mg, 36 %) and **13b-β** (22 mg, 41 %) as colorless oils.

**Data for compound 13b-α: R*_f_* =** 0.53 (PE/AcOEt 80:20); $\left[ \boldsymbol{\alpha} \right]_{\mathbf{D}}^{\mathbf{20}}$ **=** +280.0 (*c* 0.3, CHCl_3_); **^1^H NMR (500 MHz, CDCl_3_): δ** 7.80 (d, 2H, *J* = 8.2 Hz, H-Ar), 7.38 – 7.32 (m, 7H, H-Ar), 7.32 – 7.26 (m, 8H, H-Ar), 7.25 – 7.21 (m, 2H, H-Ar), 5.87 (d, 1H, *J* = 3.6 Hz, H-1), 4.89 (d, 1H, *J* = 11.0 Hz, OCH*H*Ph), 4.86 (d, 1H, *J* = 10.8 Hz, OCH*H*Ph), 4.81 – 4.76 (m, 3H, OC*H_2_*Ph), 4.69 (d, 1H, *J* = 10.8 Hz, OCH*H*Ph), 4.06 (ddd, 1H, *J* = 13.7, 4.3, 2.2 Hz, H-6’), 3.95 – 3.82 (m, 3H, H-6, H-3, H-4), 3.79 (dd, 1H, *J* = 9.3, 3.6 Hz, H-2), 3.04 (dt, 1H, *J* = 9.3, 2.2 Hz, H-5), 2.98 (dd, 1H, *J* = 11.3, 4.3 Hz, C_6_-O*H*), 2.44 (s, 3H, CH_3_-Ts); **^13^C NMR (125 MHz, CDCl_3_): δ** 144.9, 138.4, 138.1, 137.2, 135.6 (C^IV^, C-Ar), 130.2, 128.8, 128.6, 128.6, 128.4, 128.2, 128.0, 127.9, 127.9, 127.8 (CH-Ar), 81.6 (C_3_), 81.1 (C_2_), 77.3 (C_4_), 75.7, 75.5, 73.8 (O*C*H_2_Ph), 73.0 (C_1_), 59.5 (C_5_), 58.3 (C_6_), 21.8 (CH_3_-Ts); **IR (cm^-1^)**: 3527 (w), 2907 (w), 2099 (vs); **HRMS (ESI) m/z =** [M+Na]^+^ calcd for C_34_H_36_O_6_N_4_NaS 651.2248; found 651.2225.

**Data for compound 13b-β: R*_f_* =** 0.26 (PE/AcOEt 80:20); $\left[ \boldsymbol{\alpha} \right]_{\mathbf{D}}^{\mathbf{20}}$ **=** +55.0 (*c* 0.3, CHCl_3_); **^1^H NMR (500 MHz, CDCl_3_): δ** 7.73 (d, 2H, *J* = 8.2 Hz, H-Ar), 7.37 – 7.27 (m, 11H, H-Ar), 7.26 – 7.24 (m, 1H, H-Ar), 7.21 – 7.16 (m, 4H, H-Ar), 7.11 (d, 1H, *J* = 8.2 Hz, H-Ar), 5.52 (dd, 1H, *J* = 4.4, 0.4 Hz, H-1), 4.65 (d, 1H, *J* = 11.1 Hz, OCH*H*Ph), 4.61 (d, 1H, *J* = 11.1 Hz, OCH*H*Ph), 4.58 (d, 1H, *J* = 11.4 Hz, OCH*H*Ph), 4.56 – 4.50 (m, 2H, OC*H_2_*Ph), 4.48 (d, 1H, *J* = 11.4 Hz, OCH*H*Ph), 3.93 (dd, 1H, *J* = 10.0, 5.6 Hz, H-5), 3.81 (dt, 1H, *J* = 10.0, 5.0 Hz, H-6’), 3.74 (dt, 1H, *J* = 11.5, 5.6 Hz, H-6), 3.66 (dd, 1H, *J* = 7.5, 3.6 Hz, H-4), 3.54 (dd, 1H, *J* = 8.3, 4.5 Hz, H-2), 3.08 (dd, 1H, *J* = 8.3, 7.5 Hz, H-3), 2.33 (s, 3H, CH_3_-Ts); **^13^C NMR (125 MHz, CDCl_3_): δ** 144.3, 137.9, 137.7, 137.4, 135.8 (C^IV^, C-Ar), 129.8, 128.6, 128.5, 128.2, 128.0, 127.9, 127.9 (CH-Ar), 80.4 (C_2_), 80.1 (C_3_), 77.7 (C_4_), 75.5 (C_1_), 74.5, 74.1, 72.8 (O*C*H_2_Ph), 63.9 (C_6_), 60.9 (C_5_), 21.7 (CH_3_-Ts); **IR (cm^-1^)**: 3526 (w), 2905 (w), 2099 (vs); **HRMS (ESI) m/z =** [M+=Na]^+^ calcd for C_34_H_36_O_6_N_4_NaS 651.2248; found 651.2225.

###### **2,3,4-tri-*O*-benzyl-1α-cyano-*N*-tosyl-1-deoxynojirimycin 13c**

Compound **3** (50 mg, 0.08 mmol, 1.0 eq.) was treated with TMSCN (344 μL, 2.73 mmol, 32.0 eq.) and TiCl_4_ (1M/DCM, 172 μL, 0.17 mmol, 2.0 eq.) following general **procedure D** to give compound **13c** (33 mg, 66 %) as a colorless oil. **R*_f_* =** 0.39 (PE/AcOEt 80:20); $\left[ \boldsymbol{\alpha} \right]_{\mathbf{D}}^{\mathbf{20}}$ **=** -7.0 (*c* 0.3, CHCl_3_); **^1^H NMR (400 MHz, CDCl_3_): δ** 7.74 (d, 2H, *J* = 8.2 Hz, H-Ar), 7.40 – 7.27 (m, 15H, H-Ar), 7.24 – 7.19 (m, 2H, H-Ar), 5.19 (d, 1H, *J* = 5.4 Hz, H-1), 4.88 (d, 1H, *J* = 11.0 Hz, OCH*H*Ph), 4.83 – 4.71 (m, 4H, OC*H_2_*Ph), 4.66 (d, *J* = 10.5 Hz, OCH*H*Ph), 4.18 (ddd, 1H, *J* = 13.7, 4.7, 2.8 Hz, H-6’), 3.97 (ddd, 1H, *J* = 13.7, 10.7, 2.8 Hz, H-6), 3.90 – 3.80 (m, 2H, H-4, H-3), 3.76 (dd, 1H, *J* = 8.8, 5.5 Hz, H-2), 3.21 – 3.14 (m, 1H, H-5), 3.00 (dd, 1H, *J* = 10.7, 4.7 Hz, C_6_-O*H*), 2.43 (s, 3H, CH_3_-Ts); **^13^C NMR (100 MHz, CDCl_3_): δ** 145.5, 138.1, 137.9, 136.9, 134.3 (C^IV^, C-Ar), 130.4, 128.9, 128.6, 128.6, 128.5, 128.2, 128.0, 127.9, 127.9, 127.8 (CH-Ar), 114.3 (*C*N), 82.7 (C_3_), 77.8 (C_2_), 77.4 (C_4_), 75.6, 75.1, 73.6 (OC*H_2_*Ph), 61.5 (C_5_), 59.0 (C_6_), 50.2 (C_1_), 21.8 (CH_3_-Ts); **IR (cm^-1^)**: 3547 (w), 2923 (w), 2855 (w), 1723 (vw), 1702 (vw); **HRMS (ESI) m/z =** [M+H]^+^ calcd for C_35_H_37_O_6_N_2_S 613.2367; found 613.2346.

###### **1β-azido-1-deoxy-2,4-di-*O*-tosyl-*N*-tosyl-nojirimycin 13d**

Compound **7** (50 mg, 0.08 mmol, 1.0 eq.) was reacted with TMSN_3_ (85 μL, 0.64 mmol, 8.0 eq.) and TiCl_4_ (1M/DCM, 40 μL, 0.04 mmol, 0.5 eq.) following general **procedure D.** After stirring for 1h, TMSN_3_ (85 μL, 0.64 mmol, 8.0 eq.) and TiCl_4_ (1M/DCM, 40 μL, 0.04 mmol, 0.5 eq.) were added, and after 3h of stirring, TMSN_3_ (85 μL, 0.64 mmol, 8.0 eq.) and TiCl_4_ (1M/DCM, 40 μL, 0.04 mmol, 0.5 eq.) were added again to give compound **13d** (12 mg, 23 %) as a white solid. **R*_f_* =** 0.37 (PE/AcOEt 60:40); $\left[ \boldsymbol{\alpha} \right]_{\mathbf{D}}^{\mathbf{20}}$ **=** +44.3 (*c* 1.0, CHCl_3_); **^1^H NMR (500 MHz, acetone-*d*_6_): δ** 7.78 (d, 2H, *J* = 8.4 Hz, H-Ar), 7.68 (d, 2H, *J* = 8.4 Hz, H-Ar), 7.62 (d, 2H, *J* = 8.4 Hz, H-Ar), 7.54 (d, 2H, *J* = 8.4 Hz, H-Ar), 7.48 (d, 2H, *J* = 8.4 Hz, H-Ar), 7.22 (d, 2H, *J* = 8.4 Hz, H-Ar), 5.72 (d, 1H, *J* = 2.9 Hz, H-1), 5.34 (d, 1H, *J* = 5.0 Hz, C_3_-O*H*), 4.91 (dd, 1H, *J* = 3.8, 1.7 Hz, H-4), 4.59 (dd, 1H, *J* = 4.6, 2.9 Hz, H-2), 4.43 (dd, 1H, *J* = 6.5, 4.8 Hz, C_6_-O*H*), 4.16 (td, 1H, *J* = 10.1, 6.5 Hz, H-6’), 3.94 (ddd, 1H, *J* = 9.7, 5.2, 1.2 Hz, H-5), 3.80 – 3.72 (m, 2H, H-6 and H-3), 2.52 (s, 3H, CH_3_), 2.49 (s, 3H, CH_3_), 2.40 (s, 3H, CH_3_); **^13^C NMR (125 MHz, acetone-*d*_6_): δ** 146.5, 146.0, 145.1, 136.7, 134.3, 134.0 (C^IV^, C-Ar), 131.0, 130.8, 130.5, 128.8, 128.6, 128.5 (CH-Ar), 76.2 (C_2_), 75.4 (C_4_), 71.8 (C_1_), 67.8 (C_3_), 62.6 (C_6_), 58.9 (C_5_), 21.6 (CH_3_); **HRMS (ESI) m/z =** [M+Na]^+^ calcd for C_27_H_30_O_10_N_4_NaS_3_ 689.1016; found 689.1016.

###### **1α-cyano-2-C-allyl-4-O-benzyl-1,2-dideoxy-*N*-tosyl-nojirimycin 13e**

Compound **10e** (19 mg, 0.04 mmol, 1.0 eq.) was reacted with TMSCN (44 μL, 0.35 mmol, 8.0 eq.) and TiCl_4_ (1M/DCM, 22 μL, 0.02 mmol, 0.5 eq.) following general **procedure D.** After stirring for 1h, TMSCN (44 μL, 0.35 mmol, 8.0 eq.) and TiCl_4_ (1M/DCM, 22 μL, 0.02 mmol, 0.5 eq.) were added, and after 1h of stirring, TMSCN (44 μL, 0.35 mmol, 8.0 eq.) and TiCl_4_ (1M/DCM, 22 μL, 0.02 mmol, 0.5 eq.) were added again to give compound **13e** (5 mg, 25 %) as a colorless oil. **R*_f_* =** 0.41 (PE/AcOEt 60:40); $\left[ \boldsymbol{\alpha} \right]_{\mathbf{D}}^{\mathbf{20}}$ **=** -50.0 (*c* 0.4, CHCl_3_); **^1^H NMR (500 MHz, acetone-*d*_6_): δ** 7.87 (d, 2H, *J* = 8.4 Hz, H-Ar), 7.34 (d, 2H, *J* = 8.4 Hz, H-Ar), 7.30 – 7.22 (m, 3H, H-Ar), 7.20 – 7.17 (m, 2H, H-Ar), 5.96 – 5.86 (m, 1H, H-b), 5.25 – 5.15 (m, 2H, H-c’ and H-c), 5.01 (d, 1H, *J* = 4.8 Hz, H-1), 4.93 (d, 1H, *J* = 3.2 Hz, C_3_-O*H*), 4.64 (d, 1H, *J* = 11.5 Hz, OCH*H*Ph), 4.41 (d, 1H, *J* = 11.5 Hz, OCH*H*Ph), 4.13 – 4.07 (m, 1Hn H-6’), 3.98 – 3.90 (m, 2H, H-6 and C_6_-O*H*), 3.86 (dd, 1H, *J* = 5.4, 3.9 Hz, H-4), 3.66 (dd, 1H, *J* = 9.2, 3.2 Hz, H-3), 3.59 (td, 1H, *J* = 5.6, 2.8 Hz, H-5), 2.36 (s, 3H, CH_3_), 2.24 – 2.06 (m, 3H, H-2, H-a’ and H-a); **^13^C NMR (125 MHz, acetone-*d*_6_): δ** 145.3, 139.3, 136.2 (C^IV^, C-Ar), 135.7 (C_b_), 130.6, 128.9, 128.8, 128.1, 128.0 (CH-Ar), 118.6 (C_c_), 115.8 (CN), 80.9 (C_4_), 73.0 (C_3_), 72.9 (O*C*H_2_Ph), 61.9 (C_6_), 60.9 (C_5_), 48.2 (C_1_), 44.0 (C_2_), 35.0 (C_a_), 21.5 (CH_3_); **HRMS (ESI) m/z =** [M+H]^+^ calcd for C_24_H_29_O_5_N_2_S 457.1792; found 457.1783. **Xray crystallography**: CCDC 2490189 (see p 42).

###### **3-azido-1β-cyano-3-deoxy-*N*-tosyl-nojirimycin 13f**

Compound **10f** (25 mg, 0.07 mmol, 1.0 eq.) was reacted with TMSCN (73 μL, 0.59 mmol, 8.0 eq.) and TiCl_4_ (1M/DCM, 37 μL, 0.04 mmol, 0.5 eq.) following general **procedure D.** After stirring for 1h and 2h, TMSCN (147 μL, 1.18 mmol, 16.0 eq.) and TiCl_4_ (1M/DCM, 74 μL, 0.07 mmol, 1.0 eq.) to give compound **13f** (11 mg, 41 %) as a colorless oil. **R*_f_* =** 0.42 (PE/AcOEt 20:80); mp: 151.2°C; $\left[ \boldsymbol{\alpha} \right]_{\mathbf{D}}^{\mathbf{20}}$ **=** +25.1 (*c* 0.4, MeOH); **^1^H NMR (400 MHz, methanol-*d*_4_ + 0.01% CDCl_3_): δ** 7.83 (d, 2H, *J* = 8.2 Hz, H-Ar), 7.43 (d, 2H, *J* = 8.2 Hz, H-Ar), 4.64 (d, 1H, *J* = 5.9 Hz, H-1), 4.01 – 3.93 (m, 2H, H-2 and H-5), 3.80 (dd, 1H, *J* = 10.9, 6.5 Hz, H-6’), 3.75 – 3.67 (m, 2H, H-6 and H-4), 2.89 (t, 1H, *J* = 8.3 Hz, H-3), 2.45 (s, 3H, CH_3_); **^13^C NMR (100 MHz, methanol-*d*_4_ + 0.01% CDCl_3_): δ** 146.1, 137.1 (C^IV^, C-Ar), 131.0, 128.9 (CH-Ar), 119.2 (CN), 71.1 (C_2_), 70.7 (C_4_), 67.0 (C_3_), 64.2 (C_5_), 63.6 (C_6_), 51.2 (C1), 21.5 (CH_3_); **IR (cm^-1^)**: 3375 (w), 2922 (w), 2853 (w), 2229 (vw), 2110 (s); **HRMS (ESI) m/z =** [M+H]^+^ calcd for C_14_H_18_O_5_N_5_S 368.1023; found 368.1019.

###### **1,6:3,4-dianhydro-2-*O*-benzyl-*N*-tosyl-β-D-allopiperidinose 14**

To a solution of epoxide **6** (80 mg, 0.27 mmol, 1.0 eq) in dry DMF (5.38 mL, 0.05 M) under Ar atm. at 0°C were added NaH 60 % oil (21 mg, 0.54 mmol, 2.0 eq) and BnBr (48 μL, 0.40 mmol, 1.5 eq). The mixture was stirred at 0°C for 45 minutes, then neutralized with H_2_O (10 mL) and the aqueous layer was extracted with DCM (4x20 mL). The combined organic layers were dried over MgSO_4_ and concentrated under reduced pressure. The residue was purified over a chromatography column (RediSep® 4 g, PE/EtOAc 10:90 to 60:40) to afford **14** (69 mg, 66 %) as an oil. **R*_f_* =** 0.54 (PE/AcOEt 50:50); $\left[ \boldsymbol{\alpha} \right]_{\mathbf{D}}^{\mathbf{20}}$ **=** -55.0 (*c* 0.4, CHCl_3_); **^1^H NMR (500 MHz, CDCl_3_): δ** 7.86 (d, 2H, *J* = 8.2 Hz, H-Ar), 7.36 – 7.30 (m, 5H, H-Ar), 7.26 – 7.23 (m, 2H, H-Ar), 5.53 (t, 1H, *J* = 1.8 Hz, H-1), 4.76 – 4.64 (m, 3H, OC*H_2_*Ph, H-5), 3.79 (d, 1H, *J* = 7.7 Hz, H-6’), 3.62 (dd, 1H, *J* = 4.3, 1.8 Hz, H-2), 3.51 (dd, 1H, *J* = 7.7, 4.3 Hz, H-6), 3.26 (dd, 1H, *J* = 3.9, 3.0 Hz, H-4), 3.17 (td, 1H, *J* = 4.3, 1.9 Hz, H-3), 2.40 (s, 3H, CH_3_-Ts); **^13^C NMR (125 MHz, CDCl_3_): δ** 144.2, 137.5, 136.5 (C^IV^, C-Ar), 129.7, 128.6, 128.1, 128.1, 127.9 (CH-Ar), 86.3 (C_1_), 73.3 (C_2_), 71.4 (O*C*H_2_Ph), 66.7 (C_6_), 54.4 (C_5_), 50.8 (C_4_), 47.4 (C_3_), 21.8 (CH_3_-Ts); **HRMS (ESI) m/z =** [M+H]^+^ calcd for C_20_H_22_O_5_NS 388.1213; found 388.1206.

###### **2-*O*-benzyl-3-azido-3-deoxy-1,6-anhydro-*N*-tosyl-β-D-glucopiperidinose 15**

To a solution of epoxide **14** (50 mg, 0.13 mmol, 1.0 eq) in dry DMF (1.29 mL, 0.1 M) under Ar atm. at RT was added NaN_3_ (50 mg, 0.77 mmol, 6.0 eq). The reaction mixture was stirred for 36 h at 110°C, cooled down to RT, concentrated under reduced pressure and purified over a flash chromatography column (PE/EtOAc 80:20 to 60:40) to give **15** (44 mg, 80%) as an oil. **R*_f_* =** 0.56 (PE /AcOEt 60:40); $\left[ \boldsymbol{\alpha} \right]_{\mathbf{D}}^{\mathbf{20}}$ **=** -18.0 (*c* 0.1, CHCl_3_); **^1^H NMR (400 MHz, CDCl_3_): δ** 7.76 (d, 2H, *J* = 8.2 Hz, H-Ar), 7.42 - 7.31 (m, 5H, H-Ar), 7.27 (d, 2H, *J* = 8.2 Hz, H-Ar), 5.79 (d, 1H, *J* = 2.9 Hz, H-1), 4.70 (d, 1H, *J* = 11.6 Hz, OC*H*HPh), 4.61 (d, 1H, *J* = 11.6 Hz, OC*H*HPh), 4.32 - 4.24 (m, 1H, H-5), 3.86 (d, 1H, *J* = 8.2 Hz, H-6’), 3.71 (s, 1H, H-3), 3.67 - 3.56 (m, 2H, H-4, H-2), 3.05 (dd, 1H, *J* = 8.2, 5.5 Hz, H-6), 2.89 (d, 1H, *J* = 11.9 Hz, C_4_-OH), 2.42 (s, 3H, CH_3_-Ts); **^13^C NMR (100 MHz, CDCl_3_): δ** 144.9, 136.6, 135.5 (C^IV^, C-Ar), 130.1, 128.8, 128.5, 128.2, 127.8 (CH-Ar), 87.1 (C_1_), 75.6 (C_2_), 72.2 (O*C*H_2_Ph), 70.2 (C_4_), 65.5 (C_6_), 62.1 (C_3_), 60.9 (C_5_), 21.8 (CH_3_-Ts); **IR (cm^-1^)**: 3529 (w), 2905 (w), 2099 (vs), 1597 (w); **HRMS (ESI) m/z =** [M+Na]^+^ calcd for C_20_H_22_O_5_N_4_NaS 453.1203; found 453.1198.

###### **1,6-anhydro-3-azido-2-*O*-benzyl-3,4-dideoxy-4-fluoro-*N*-tosyl-β-D-galactopiperidinose 16**

Compound **15** (126 mg, 0.29 mmol, 1.0 eq.) was treated with Tf_2_O (110 μL, 0.67 mmol, 2.3 eq.) following general **procedure B** to give the corresponding triflate (157 mg, quant.) as a yellow oil, which

was used in the following step without purification.

To a solution of this triflate in dry DCM (2.93 mL, 0.1M) under Ar atm. at RT was added TBAF.3H_2_O (277 mg, 0.88 mmol, 3.0 eq). The reaction mixture was stirred at RT for 15 h, when TLC showed total consumption of the starting triflate. The reaction mixture was diluted with H_2_O (10 mL) and the aqueous layer was extracted with DCM (4x15 mL). The combined organic layers were dried over MgSO_4_ and concentrated under reduced pressure. The residue was purified over a flash chromatography column (RediSep® 4 g, PE/EtOAc 99:1 to 60:40) to afford **16** (89 mg, 71 % over 2 steps) as a colorless oil. **R*_f_* =** 0.33 (PE/AcOEt 85:15); $\left[ \boldsymbol{\alpha} \right]_{\mathbf{D}}^{\mathbf{20}}$ **=** -13.0 (*c* 0.5, CHCl_3_); **^1^H NMR (400 MHz, CDCl_3_): δ** 7.72 (d, 2H, *J* = 8.2 Hz, H-Ar), 7.37 – 7.20 (m, 7H, H-Ar), 5.61 (t, 1H, *J* = 2.9 Hz, H-1), 4.93 (dt, 1H, *J* = 45.7, 5.0 Hz, H-4), 4.63 (d, 1H, *J* = 11.5 Hz, OCH*H*Ph), 4.52 (d, 1H, *J* = 11.5 Hz, OCH*H*Ph), 4.36 (q, 1H, *J* = 4.4 Hz, H-5), 4.15 (d, 1H, *J* = 8.2 Hz, H-6’), 3.98 (d, 1H, *J* = 5.7 Hz, H-3), 3.66 (br.s, 1H, H-2), 2.98 (dd, 1H, *J* = 8.2, 4.4 Hz, H-6), 2.38 (s, 3H, CH_3_-Ts); **^13^C NMR (100 MHz, CDCl_3_): δ** 145.0, 136.8, 135.5 (C^IV^, C-Ar), 130.1, 128.8, 128.5, 128.2, 127.9 (CH-Ar), 86.7 (d, *J* = 192.1 Hz, C_4_), 86.4 (C_1_), 78.7 (d, *J* = 3.2 Hz, C_2_), 72.4 (O*C*H_2_Ph), 64.1 (C_6_), 59.9 (d, *J* = 14.8 Hz, C_3_), 56.8 (d, *J* = 29.9 Hz, C_5_), 21.8 (CH_3_-Ts); **^19^F NMR (375 MHz, CDCl_3_): δ** -194.6 (s); **IR (cm^-1^)**: 2923 (w), 2853 (w), 2111 (s), 1320 (s); **HRMS (ESI) m/z =** [M+H]^+^ calcd for C_20_H_22_O_4_N_4_FS 433.1340; found 433.1332.

###### **1β-*C*-allyl-3-azido-2-*O*-benzyl-1,3,4-tri-deoxy-4-fluoro-*N*-tosylgalactonojirimycin 17**

Compound **16** (43 mg, 0.10 mmol, 1.0 eq.) was reacted with TMS-allyl (63 μL, 0.40 mmol, 4.0 eq.) and TiCl_4_ (1M/DCM, 49 μL, 0.05 mmol, 0.5 eq.) following general **procedure D** to give compound **17** (13 mg, 39 %) as a colorless oil. **R*_f_* =** 0.31 (PE/AcOEt 80:20); $\left[ \boldsymbol{\alpha} \right]_{\mathbf{D}}^{\mathbf{20}}$ **=** +44.5 (*c* 0.4, CHCl_3_); **^1^H NMR (400 MHz, CDCl_3_): δ** 7.70 (d, 2H, *J* = 8.2 Hz, H-Ar), 7.43 – 7.28 (m, 5H, H-Ar), 7.11 (d, 2H, *J* = 8.2 Hz, H-Ar), 5.86 (dq, 1H, *J* = 9.9, 7.1 Hz, H-b), 5.27 – 5.11 (m, 2H, H-c’, H-c), 4.86 (ddd, 1H, *J* = 49.9, 5.6, 2.1 Hz, H-4), 4.60 (d, 1H, *J* = 11.2 Hz, OCH*H*Ph), 4.48 (d, 1H, *J* = 11.2 Hz, OCH*H*Ph), 4.40 (t, 1H, *J* = 8.3 Hz, H-1), 4.05 – 3.79 (m, 3H, H-6’, H-6, H-5), 3.64 (d, 1H, *J* = 7.0 Hz, H-2), 2.84 (dd, 1H, *J* = 28.3, 6.5 Hz, H-3), 2.61 – 2.42 (m, 3H, H-a’, H-a, C_6_-O*H*), 2.36 (s, 3H, CH_3_-Ts); **^13^C NMR (100 MHz, CDCl_3_): δ** 144.3, 137.0, 135.6 (C^IV^, C-Ar), 134.3 (C_b_), 129.8, 128.7, 128.3, 128.1, 127.9 (CH-Ar), 119.3 (C_c_), 88.1 (d, *J* = 183.1 Hz, C_4_), 78.8 (d, *J* = 5.0 Hz, C_2_), 72.0 (O*C*H_2_Ph), 62.0 (C_6_), 61.8 (d, *J* = 8.3 Hz, C_3_), 58.7 (C_1_), 57.9 (d, *J* = 21.8 Hz, C_5_), 39.7 (d, *J* = 6.1 Hz, C_a_), 21.7 (CH_3_-Ts); **^19^F NMR (375 MHz, CDCl_3_): δ** -203.5 (s); **HRMS (ESI) m/z =** [M+H]^+^ calcd for C_23_H_28_O_4_N_4_FS 475.1810; found 475.1793.

###### **1,6-anhydro-2-azido-4-*O*-benzyl-2-deoxy-3-*O*-[(1*S*)-1-methoxycarboxyethyl]-*N*-tosyl-β-D-glucopiperidinose 19**

To a solution of alcohol **10c** (70 mg, 0.163 mmol, 1.0 eq.) in dry THF (3 mL), at RT under Ar, was added the freshly prepared methyl (*S*)-2-(((trifluoromethyl)sulfonyl)oxy)propanoate **18**^[1]^ (192 mg, 0.815 mmol, 5.0 eq.). The solution was cooled down to 0°C, then NaH (18 mg, 0.489 mmol, 3.0 eq.) was added. The mixture was stirred at 0°C for 10 minutes and then at RT for 2.5 h., then diluted with DCM (10 mL) and quenched with water (15 mL). The aqueous phase was extracted with DCM (3 x 15 mL), the combined organic layers were dried over MgSO_4_ and concentrated under reduced pressure. The crude was purified over a chromatography column (PE/EtOAc 80:20 then 70:30) to afford **19** (62 mg, 74 %) as a white solid. **R*_f_* =** 0.47 (PE/AcOEt 70:30); mp: 100.9°C; $\left[ \boldsymbol{\alpha} \right]_{\mathbf{D}}^{\mathbf{20}}$ **=** +9.9 (*c* 0.8, CHCl_3_); **^1^H NMR (400 MHz, CDCl_3_): δ** 7.77 (d, 2H, *J* = 8.3 Hz, H-Ar), 7.30 - 7.18 (m, 7H, H-Ar), 5.63 (d, 1H, *J* = 2.4 Hz, H-1), 4.51 (d, 1H, *J* = 11.9 Hz, OCH*H*Ph), 4.37 (d, 1H, *J* = 11.9 Hz, OCH*H*Ph), 4.34 (d, 1H, *J* = 5.8 Hz, H-5), 3.89 (q, 1H, *J* = 6.8 Hz, H-a), 3.75 (d, 1H, *J* = 7.8 Hz, H-6’), 3.67 (s, 3H, CO_2_CH_3_), 3.62 (s, 1H, H-2), 3.45 (s, 1H, H-3), 3.33 (s, 1H, H-4), 3.25 (dd, 1H, *J* = 7.8, 6.0 Hz, H-6), 2.33 (s, 3H, CH_3_-Ts), 1.25 (d, 3H, *J* = 6.9 Hz, CH_3_-chain); **^13^C NMR (125 MHz, CDCl_3_): δ** 172.9 (C=O), 144.5, 137.0, 135.6 (C^IV^, C-Ar), 129.7, 128.5, 128.0, 127.8, (CH-Ar), 87.6 (C_1_), 78.5 (C_3_), 77.1 (C_4_), 74.7 (C_a_), 70.7 (O*C*H_2_Ph), 65.0 (C_6_), 62.7 (C_2_), 56.8 (C_5_), 52.2 (CH_3_-CO_2_Me), 21.7 (CH_3_-Ts), 18.8 (CH_3_-chain); **HRMS (ESI) m/z =** [M+Na]^+^ calcd for C_24_H_28_N_4_O_7_SNa 539.1571; found 539.1556; **X-ray crystallography**: CCDC 2447464 (see p 44).

###### **2-acetamido-1,6-anhydro-4-*O*-benzyl-2-deoxy-3-*O*-[(1*S*)-1-methoxycarboxyethyl]-*N*-tosyl-β-D-glucopiperidinose 20**

To a solution of azide **19** (55 mg, 0.106 mmol, 1.0 eq.) in a mixture of THF (2 mL) and NH_4_OH (200 μL), at RT under Ar, was added supported triphenylphosphine (1.5 mmol/g, 355 mg, 0.532 mmol, 5.0 eq.). The suspension was stirred at 80°C for 2 h., then cooled down to RT, filtrated on a pad of celite and concentrated under reduced pressure. The crude amine was diluted in pyridine (500 μL) and treated with acetic anhydride (500 μL) for 2h at RT, then concentrated and co-evaporated twice with toluene. The crude was purified over a chromatography column (PE/EtOAc 50:50 to 30:70) to afford **20** (30 mg, 53 % over 2 steps) as an oil. **R*_f_* =** 0.27 (PE/AcOEt 40:60); $\left[ \boldsymbol{\alpha} \right]_{\mathbf{D}}^{\mathbf{20}}$ **=** -25.1 (*c* 1.0, CHCl_3_); **^1^H NMR (500 MHz, CDCl_3_): δ** 7.74 (d, 2H, *J* = 8.3 Hz, H-Ar), 7.39 - 7.34 (m, 5H, H-Ar), 7.27 (d, 2H, *J* = 8.2 Hz, H-Ar), 6.04 (d, 1H, *J* = 9.0 Hz, NH), 5.50 (d, 1H, *J* = 3.0 Hz, H-1), 4.81 (d, 1H, *J* = 11.6 Hz, OCH*H*Ph), 4.52 (d, 1H, *J* = 11.6 Hz, OCH*H*Ph), 4.48 (dd, 1H, *J* = 2.2, 3.0 Hz, H-5), 4.30 (m, 1H, H-2), 4.16 (q, 1H, *J* = 6.8 Hz, H-a), 3.90 (d, 1H, *J* = 7.9 Hz, H-6’), 3.71 (s, 3H, CO_2_CH_3_), 3.53 (t, 1H, *J* = 1.4 Hz, H-4), 3.39 (s, 1H, H-3), 2.92 (dd, 1H, *J* = 7.8, 5.9 Hz, H-6), 2.41 (s, 3H, CH_3_-Ts), 1.88 (s, 3H, CH_3_-NHAc), 1.31 (d, 3H, *J* = 6.8 Hz, CH_3_-chain); **^13^C NMR (125 MHz, CDCl_3_): δ** 172.9 (C=O), 169.5 (C=O), 144.6, 137.1, 135.4 (C^IV^, C-Ar), 129.9, 128.6, 128.1, 127.8, 127.6 (CH-Ar), 88.2 (C_1_), 77.3 (C_4_), 77.2 (C_3_), 73.9 (C_a_), 70.7 (O*C*H_2_Ph), 65.1 (C_6_), 57.0 (C_5_), 52.2 (CH_3_-CO_2_Me), 50.6 (C_2_), 23.2 (CH_3_-NHAc), 21.6 (CH_3_-Ts), 18.0 (CH_3_-chain); **IR (cm^-1^)**: 2904 (w), 1735 (s), 1676 (s), 1505 (s), 1350 (s), 1163 (s); **HRMS (ESI) m/z =** [M+Na]^+^ calcd for C_26_H_32_N_2_O_8_SNa 555.1772; found 555.1763.

###### **2-acetamido-1,6-anhydro-3-*O*-[(1*S*)-1-carboxyethyl]-2-deoxy-*N*-tosyl-β-D-glucopiperidinose 21**

To a solution of ester **20** (12 mg, 0.022 mmol, 1.0 eq.) in a mixture of THF/dioxane (2/1, 300:150 μL), at 0°C under Ar, was added a solution of LiOH (1M, 66 μL, 0.066 mmol, 3.0 eq.). The mixture was stirred at 0°C for 30 minutes then at RT for 5h and concentrated under reduced pressure. The crude carboxylic acid was directly used without purification for the final hydrogenolysis step. The crude was diluted in MeOH (LCMS grade, 1 mL) and Pd(OH)_2_/C (25 mg) was added. The mixture was stirred at RT under H_2_ atmosphere for 20h, then filtrated on a pad of celite (previously washed successively with 1M HCl, H_2_O, MeOH), and concentrated under reduced pressure to afford **21** (9.6 mg, quant. over 2 steps) as a colorless oil. **R*_f_* =** 0.32 (AcOEt/MeOH 60:40); $\left[ \boldsymbol{\alpha} \right]_{\mathbf{D}}^{\mathbf{20}}$ **=** -6.0 (*c* 1.0, CH_3_OH); **^1^H NMR (500 MHz, methanol-*d*_4_): δ** 8.54 (s, 1H, NH), 7.77 (d, 2H, *J* = 8.2 Hz, H-Ar), 7.39 (d, 2H, *J* = 8.1 Hz, H-Ar), 5.50 (d, 1H, *J* = 2.3 Hz, H-1), 4.27 (d, 1H, *J* = 4.7 Hz, H-5), 4.11 (q, 1H, *J* = 6.8 Hz, H-a), 4.00 (t, 1H, *J* = 7.8 Hz, H-2), 3.80 (d, 1H, *J* = 7.8 Hz, H-6’), 3.74 (t, 1H, *J* = 2.4 Hz, H-4), 3.29 (m, 1H, H-3), 2.86 (dd, 1H, *J* = 7.9, 5.9 Hz, H-6), 2.44 (s, 3H, CH_3_-Ts), 1.95 (s, 3H, CH_3_-NHAc), 1.28 (d, 3H, *J* = 6.8 Hz, CH_3_-chain); **^13^C NMR (125 MHz, methanol-*d*_4_): δ** 181.1 (C=O, COOH), 172.9 (C=O, NHAc), 146.1, 137.0 (C^IV^, C-Ar), 130.9, 129.0 (CH-Ar), 89.3 (C_1_), 79.9 (C_3_), 77.6 (C_a_), 72.9 (C_4_), 66.7 (C_6_), 62.6 (C_5_), 55.4 (C_2_), 22.6 (CH_3_-NHAc), 21.5 (CH_3_-Ts), 19.6 (CH_3_-chain); **HRMS (ESI) m/z =** [M+Na]^+^ calcd for C_18_H_24_N_2_O_8_SNa 451.1146; found 451.1138.

**4. Crystallographic data**

Crystallographic data for **4**

Deposition Number 2447469

----------------------------------------------------------------

Summary of Data – Deposition Number 2447469

----------------------------------------------------------------

###### Compound Name: 1,6-anhydro-*N*-tosyl-β-D-glucopiperidinose

TITL YB_DY_1_188_0m_a.res in P2(1)

yb_dy_1_188_0m_a.res

created by SHELXL-2019/3 at 16:29:15 on 27-Jun-2023

----------------------------------------------------------------

Crystallographic data for **6**

Deposition Number 2487464

----------------------------------------------------------------

Summary of Data – Deposition Number 2487464

----------------------------------------------------------------

Compound Name: 1,6:2,3-dianhydro-*N*-tosyl-β-D-allopiperidinose

Data Block Name: data_yb_dy_2_56fa_0m_a
Unit Cell Parameters: a 6.5477(4) b 9.7391(5) c 21.4277(12) P212121
---------------------------------------------------------------

Crystallographic data for **10b**

Deposition Number 2447468

----------------------------------------------------------------

Summary of Data – Deposition Number 2447468

----------------------------------------------------------------

###### Compound Name: 2,6:3,4-dianhydro-1-azido-1-deoxy-*N*-tosyl-α-D-altropiperidinose

data_yb_dy_2_187_f2_0m_a

_audit_creation_date	2024-05-23

_audit_creation_method

Olex2 1.5

(compiled 2024.02.16 svn.r378c4104 for OlexSys, GUI svn.r6928)

----------------------------------------------------------------

Crystallographic data for **10g**

Deposition Number 2447466

----------------------------------------------------------------

Summary of Data – Deposition Number 2447466

----------------------------------------------------------------

###### Compound Name: 1,6-anhydro-3-deoxy-3-methyl-*N*-tosyl-β-D-glucopiperidinose

data_yb_dy_3_160f3_0m_a

_audit_creation_date	2025-03-24

_audit_creation_method

;

Olex2 1.5

(compiled 2025.03.04 svn.red2d20d0 for OlexSys, GUI svn.r7188)

----------------------------------------------------------------

Crystallographic data for **10h**

Deposition Number 2447465

----------------------------------------------------------------

Summary of Data – Deposition Number 2447465

----------------------------------------------------------------

###### Compound Name: 3-allyl-1,6-anhydro-3-deoxy-*N*-tosyl-β-D-glucopiperidinose

data_yb_dy_3_165f2_0m_a

_audit_creation_date	2025-04-03

_audit_creation_method

;

Olex2 1.5

(compiled 2025.03.04 svn.red2d20d0 for OlexSys, GUI svn.r7188)

----------------------------------------------------------------

Crystallographic data for **10i**

Deposition Number 2447465

----------------------------------------------------------------

Summary of Data – Deposition Number 2447465

----------------------------------------------------------------

###### Compound Name: 1,6-anhydro-3-bromo-3-deoxy-*N*-tosyl-β-D-glucopiperidinose

data_yb_dy_3_165f2b_0m_a

_audit_creation_date	2025-04-03

_audit_creation_method

;

Olex2 1.5

(compiled 2025.03.04 svn.red2d20d0 for OlexSys, GUI svn.r7188)

----------------------------------------------------------------

Crystallographic data for **10k**

Deposition Number 2447467

----------------------------------------------------------------

Summary of Data – Deposition Number 2447467

----------------------------------------------------------------

###### Compound Name: 1,6:2,3-dianhydro-4-deoxy-4-fluoro-N-tosyl-β-D-gulopiperidinose

data_yb_dy_3_23f1_0m_a

_audit_creation_date	2024-10-04

_audit_creation_method

;

Olex2 1.5

(compiled 2024.02.16 svn.r378c4104 for OlexSys, GUI svn.r6928)

----------------------------------------------------------------

Crystallographic data for **13e**

Deposition Number 2490189

----------------------------------------------------------------

Summary of Data – Deposition Number 2490189

###### Compound Name: 1α-cyano-2-C-allyl-4-O-benzyl-1,2-dideoxy-*N*-tosyl-nojirimycin

Crystallographic data for **19**

Deposition Number 24474664

----------------------------------------------------------------

Summary of Data – Deposition Number 2447464

----------------------------------------------------------------

###### Compound Name: 1,6-anhydro-2-azido-4-*O*-benzyl-2-deoxy-3-*O*-[(1*S*)-1-methoxycarboxyethyl]-*N*-tosyl-β-D-glucopiperidinose

data_yb_jd_569_0m_a

_audit_creation_date	2025-04-14

_audit_creation_method

;

Olex2 1.5

(compiled 2025.04.05 svn.rf049d14d for OlexSys, GUI svn.r7205)

----------------------------------------------------------------

**5. Copies of ^1^H, ^19^F and ^13^C NMR spectra**

^1^H NMR spectrum (CDCl_3_, 400 MHz) of compound **2**

^13^C {1H} NMR spectrum (CDCl_3_, 100 MHz) of compound **2**

^1^H NMR spectrum (CDCl_3_, 400 MHz) of compound **3**

^13^C {1H} NMR spectrum (CDCl_3_, 100 MHz) of compound **3**

^1^H NMR spectrum (CD_3_OD, 400 MHz) of compound **4**

^13^C {1H} NMR spectrum (CD_3_OD, 100 MHz) of compound **4**

^1^H NMR spectrum (CDCl_3_, 400 MHz) of compound **5**

^13^C {1H} NMR spectrum (CDCl_3_, 100 MHz) of compound **5**

^1^H NMR spectrum (CDCl_3_, 400 MHz) of compound **6**

^13^C {1H} NMR spectrum (CDCl_3_, 100 MHz) of compound **6**

^1^H NMR spectrum (CDCl_3_, 400 MHz) of compound **7**

^13^C {1H} NMR spectrum (CDCl_3_, 100 MHz) of compound **7**

^1^H NMR spectrum (CDCl_3_, 400 MHz) of compound **8**

^13^C NMR spectrum (CDCl_3_, 100 MHz) of compound **8**

^1^H NMR spectrum (CDCl_3_, 400 MHz) of compound **9**

^13^C {1H} NMR spectrum (CDCl_3_, 100 MHz) of compound **9**

^1^H NMR spectrum (CDCl_3_, 400 MHz) of compound **10a**

^13^C {1H} NMR spectrum (CDCl_3_, 100 MHz) of compound **10a**

^1^H NMR spectrum (CDCl_3_, 500 MHz) of compound **10b**

^13^C {1H} NMR spectrum (CDCl_3_, 125 MHz) of compound **10b**

^1^H NMR spectrum (CDCl_3_, 400 MHz) of compound **10c**

^13^C {1H} NMR spectrum (CDCl_3_, 100 MHz) of compound **10c**

^1^H NMR spectrum (CDCl_3_, 400 MHz) of compound **10d**

^13^C {1H} NMR spectrum (CDCl_3_, 100 MHz) of compound **10d**

^19^F NMR spectrum (CDCl_3_, 375 MHz) of compound **10d**

^1^H NMR spectrum (CDCl_3_, 400 MHz) of compound **10e**

^13^C {1H} NMR spectrum (CDCl_3_, 100 MHz) of compound **10e**

^1^H NMR spectrum (CD_3_OD, 500 MHz) of compound **10f**

^13^C {1H} NMR spectrum (CD_3_OD, 125 MHz) of compound **10f**

^1^H NMR spectrum (CDCl_3_, 500 MHz) of compound **10g**

^13^C {1H} NMR spectrum (CDCl_3_, 125 MHz) of compound **10g**

^1^H NMR spectrum (CDCl_3_, 400 MHz) of compound **10h**

^13^C {1H} NMR spectrum (CDCl_3_, 100 MHz) of compound **10h**

^1^H NMR spectrum (CD_3_OD, 400 MHz) of compound **10i**

^13^C {1H} NMR spectrum (CD_3_OD, 100 MHz) of compound **10i**

^1^H NMR spectrum (CDCl_3_, 400 MHz) of compound **10j**

^13^C {1H} NMR spectrum (CDCl_3_, 100 MHz) of compound **10j**

^1^H NMR spectrum (CDCl_3_, 400 MHz) of compound **10k**

^13^C {1H} NMR spectrum (CDCl_3_, 100 MHz) of compound **10k**

^19^F NMR spectrum (CDCl_3_, 376 MHz) of compound **10k**

^1^H NMR spectrum (CDCl_3_, 400 MHz) of compound **10l**

^13^C {1H} NMR spectrum (CDCl_3_, 100 MHz) of compound **10l**

^1^H NMR spectrum (CDCl_3_, 500 MHz) of compound **10m**

^13^C {1H} NMR spectrum (CDCl_3_, 125 MHz) of compound **10m**

^1^H NMR spectrum (Acetone-*d*_6_, 400 MHz) of compound **10n**

^13^C {1H} NMR spectrum (Acetone-*d*_6_, 100 MHz) of compound **10n**

^19^F NMR spectrum (Acetone-*d*_6_, 376 MHz) of compound **10n**

^1^H NMR spectrum (CDCl_3_, 500 MHz) of compound **10o**

^13^C {1H} NMR spectrum (CDCl_3_, 125 MHz) of compound **10o**

^1^H NMR spectrum (CDCl_3_, 400 MHz) of compound **11a**

^13^C {1H} NMR spectrum (CDCl_3_, 100 MHz) of compound **11a**

^1^H NMR spectrum (CDCl_3_, 400 MHz) of compound **11b**

^13^C {1H} NMR spectrum (CDCl_3_, 100 MHz) of compound **11b**

^1^H NMR spectrum (CDCl_3_, 400 MHz) of compound **11c**

^13^C {1H} NMR spectrum (CDCl_3_, 100 MHz) of compound **11c**

^1^H NMR spectrum (CDCl_3_, 500 MHz) of compound **11d**

^13^C {1H} NMR spectrum (CDCl_3_, 125 MHz) of compound **11d**

^1^H NMR spectrum (CDCl_3_, 400 MHz) of compound **12b**

^13^C {1H} NMR spectrum (CDCl_3_, 100 MHz) of compound **12b**

^1^H NMR spectrum (CDCl_3_, 500 MHz) of compound **12c**

^13^C {1H} NMR spectrum (CDCl_3_, 125 MHz) of compound **12c**

^1^H NMR spectrum (CDCl_3_, 500 MHz) of compound **12d**

^13^C {1H} NMR spectrum (CDCl_3_, 125 MHz) of compound **12d**

^1^H NMR spectrum (Acetone-*d*_6_, 400 MHz) of compound **13a-α**

^13^C {1H} NMR spectrum (Acetone-*d*_6_, 100 MHz) of compound **13a-α**

^1^H NMR spectrum (Acetone-*d*_6_, 400 MHz) of compound **13a-β**

^13^C {1H} NMR spectrum (Acetone-*d*_6_, 100 MHz) of compound **13a-β**

^1^H NMR spectrum (CDCl_3_, 500 MHz) of compound **13b-α**

^13^C {1H} NMR spectrum (CDCl_3_, 125 MHz) of compound **13b-α**

^1^H NMR spectrum (CDCl_3_, 500 MHz) of compound **13b-β**

^13^C {1H} NMR spectrum (CDCl_3_, 125 MHz) of compound **13b-β**

^1^H NMR spectrum (CDCl_3_, 400 MHz) of compound **13c**

^13^C {1H} NMR spectrum (CDCl_3_, 100 MHz) of compound **13c**

^1^H NMR spectrum (Acetone-*d*_6_, 500 MHz) of compound **13d**

^13^C {1H} NMR spectrum (Acetone-*d*_6_, 125 MHz) of compound **13d**

^1^H NMR spectrum (Acetone-*d*_6_, 500 MHz) of compound **13e**

^13^C {1H} NMR spectrum (Acetone-*d*_6_, 125 MHz) of compound **13e**

^1^H NMR spectrum (CD_3_OD, 400 MHz) of compound **13f**

^13^C {1H} NMR spectrum (CD_3_OD, 100 MHz) of compound **13f**

^1^H NMR spectrum (CDCl_3_, 500 MHz) of compound **14**

^13^C {1H} NMR spectrum (CDCl_3_, 125 MHz) of compound **14**

^1^H NMR spectrum (CDCl_3_, 400 MHz) of compound **15**

^13^C {1H} NMR spectrum (CDCl_3_, 100 MHz) of compound **15**

^1^H NMR spectrum (CDCl_3_, 400 MHz) of compound **16**

^13^C {1H} NMR spectrum (CDCl_3_, 100 MHz) of compound **16**

^19^F NMR spectrum (CDCl_3_, 375 MHz) of compound **16**

^1^H NMR spectrum (CDCl_3_, 400 MHz) of compound **17**

^13^C {1H} NMR spectrum (CDCl_3_, 100 MHz) of compound **17**

^19^F NMR spectrum (CDCl_3_, 375 MHz) of compound **17**

^1^H NMR spectrum (CDCl_3_, 400 MHz) of compound **19**

^13^C {1H} NMR spectrum (CDCl_3_, 125 MHz) of compound **19**

^1^H NMR spectrum (CDCl_3_, 500 MHz) of compound **20**

^13^C {1H} NMR spectrum (CDCl_3_, 125 MHz) of compound **20**

^1^H NMR spectrum (CD_3_OD, 500 MHz) of compound **21**

^13^C {1H} NMR spectrum (CD_3_OD, 125 MHz) of compound **21**

**6. NMR conformational analysis of piperidines 13a-f and 17**

***General Procedure:***

The conformational analysis was carried out by combining experimental NMR data with molecular modelling calculations. Initially, the relevant vicinal coupling constants were extracted from the experimental ^1^H NMR spectra. The corresponding molecular structures were then constructed using Maestro/MacroModel, followed by geometry minimization and a conformational search employing the OPLS4 force field.

For the lowest-energy conformers identified, the corresponding scalar coupling constants were calculated using the MSPIN program (Bruker). These calculated values were subsequently compared with the experimental data. Based on this comparison, the ring geometry was assigned either to a single predominant conformation or, when no individual structure adequately reproduced the experimental results, to an equilibrium involving two or more conformers.

***Conformational analysis of piperidines 13a-f and 17:***

- Compounds **13b** (**α** and **β**)

For the α‑epimer **13b**α in CDCl3, the *J*_2,3_, *J*_3,4_, and *J*_4,5_ coupling constant values (9.0, 8.9, and 9.0 Hz, respectively) correspond to axial–axial relationships, characteristic of the canonical ^4^*C*_1_ chair conformation. The *J*_1,2_ coupling constant (3.6 Hz) is typical of the equatorial–axial arrangement expected for the α‑anomer. Interestingly, the OH‑6 proton displays well defined coupling constants (4.1 Hz and 11.4 Hz) to the H‑6 protons, indicating that it is in an anti-orientation respect to one of them. This presentation is consistent with the existence of a hydrogen bond formed between OH‑6 and one oxygen atoms at the tosyl (Ts) group. The *J*_5,6a_ and *J*_5,6’_ coupling values, both around 2.2 Hz, agree with a very predominant *gauche–gauche (gg)* arrangement around the hydroxy-methyl group. NOE correlations between the tosyl aromatic protons (2/6‑Ts) and H‑1 (weak), H‑5 (weak), and H‑6 proS (strong) further support this arrangement and define the orientation of the aromatic ring relative to the pyranose framework. Altogether, these data indicate a well‑organized three‑dimensional arrangement of substituents in **13b**α, stabilized by stereoelectronic and hydrogen‑bonding interactions. The Macromodel derived structure for this ^4^*C*_1_ chair is shown in Figure 1 (left).

For **13b**β, NMR analysis also provides clear insights into its preferred conformation, somehow different to the α analogue. The *J*_2,3_ and *J*_3,4_ values (both close to 8 Hz) remain large suggesting near axial–axial relationships, consistent with the benzyloxy substituents adopting pseudo-equatorial positions. However, the *J* _1,2_ (4.4 Hz) and *J* _4,5_ (3.6 Hz) encode *gauche*-like relationships (axial-equatorial or related arrangements). A small, but measurable long‑range *J*_1,5_ coupling (0.4–0.5 Hz) indicates a W‑type arrangement, consistent with both protons displaying pseudo-equatorial orientations. These findings point to a distortion of the chair conformation in the vicinity of the nitrogen atom. Unlike the α‑analogue, the OH‑6 proton appears now as a broad signal, suggesting motion around the H6-O6 bond, consistent with the absence of stabilizing intramolecular hydrogen bonding. Moreover, Consistent with this, the *J*_5,6a_ and *J*_5,6’_ coupling values, (5.1 and 5.6 Hz) reflect a dynamic equilibrium between the *gg* and *gt* conformations of the C5–C6 hydroxymethyl bond. NOE experiments provide further support to deduce the conformation. The tosyl aromatic protons (2/6‑Ts) show NOEs with all H‑1, H‑5, and H‑3, confirming that the Ts group lies on the non-polar face of the β‑conformer. Additional NOEs between H‑6/H‑6′ and both H‑2 and H‑4 define the spatial relationships around the C5–C6 region. Taken together, the scalar couplings, long‑range interactions, hydroxyl mobility, and NOE data support the ^2^*S*_N_ ring conformation as the predominant solution geometry of **13b**β. Indeed, the expected (MSPIN-calculated J-values) for this ^2^*S*_N_ geometry are in good agreement with the experimental values (Table 1). The Macromodel derived structure for this geometry, which is indeed a local minimum for this force field, in shown in Figure 1 (right).

|  |  |
| --- | --- |

Figure 1. Left: the proposed major ^4^*C*_1_ conformer for **13b**α. Right: the proposed major ^2^*S*_N_ conformer for **13b**β; the orientation of the aromatic ring satisfactorily explained the observed shielding of H1, H3, and H5. The OPLS4 force field as implemented in Macromodel within the Maestro suite of programmes was employed.

Table 1. The coupling constant values (*J,* Hz) measured for **13b**α and **13b**β at 400 MHz in CDCl3. For **13b**α, the large *J*_2,3_, *J*_3,4_, and *J*_4,5_ values point out the existence of a highly predominant ^4^*C*_1_ chair. For **13b**β, in contrast, the observed values satisfactorily match those expected for a ^2^*S*_N_ geometry.

|  |  | *J*_1,2_ | *J*_2,3_ | *J*_3,4_ | *J*_4,5_ | *J*_5,6_ | *J*_5,6’_ | others | Conformer |
| --- | --- | --- | --- | --- | --- | --- | --- | --- | --- |
| **13b**α | Exp (Hz) | 3.6 | 9.0 | 8.9 | 9.0 | 2.2 | 2.4 | 4.3/11.4 (OH-6) | ^4^*C*_1_ |
| **13b**β | Exp (Hz) | 4.4 | 8.3 | 7.5 | 3.6 | 5.6 | 5.0 | ^4^J_15_=0.5 (W-type coupling) | ^2^*S*_N_ (major) |
|  | J*_calc_* with MSPIN for a ^2^*S*_N_ geometry (Hz) | 6.0 | 9.8 | 8.0 | 2.0 |  |  |  |  |

Additionally, the comparison of the observed chemical shifts for both isomers provided an additional diagnostic criterion fully consistent with the structural picture derived from the *J/NOE* analyses (Figure 2). The proton signals of **13b**β are systematically more shielded than those in **13b**α, due to the orientation of the aromatic ring on the apolar face, which places most of the protons under its shielding cone. The only exception is H‑5, whose chemical shift in **13b**α is already unusually shielded. Nevertheless, this fact can be readily explained by the lateral positioning of the aromatic ring relative to this proton (Figure 1 right). This observation further reinforces the conformational models proposed for both anomers.

Figure 2. The dramatic chemical shift differences observed in the ^1^H NMR spectra between the **13b**α (top) and **13b**β (bottom) epimers can be explained by interactions of the aromatic moiety with the protons at the bottom face of the six-membered ring. The representation of **13b**β at the top spectrum also shows the hydrogen bond between OH-6 with the oxygen at the N-substituent. The OBn groups have been changed to methyl moieties for clarity.

- Compound **13e**:

Notably, the X‑ray crystallographic structure of **13e**α shows a ^5^*S*_1_ conformation in the solid state, while the analysis of the solution‑phase J coupling constants reveals the presence of a 60:40 conformational equilibrium between the crystallographic ^5^*S*_1_ skew boat and the ^4^*C*_1_ chair (Table 2).

Table 2. The observed coupling constant values (*J,* Hz) measured for the **13e**α at 500 MHz in acetone compared to those expected (using MSpin software) for the experimental X-ray crystallographic structure. There are notable differences between the experimental and expected values. Indeed, the fit to the experimental values is highly improved when considering a conformational equilibrium between the ^4^*C*_1_ chair and the ^5^*S*_1_ X-Ray geometry (right-hand column).

| **13e**α | *J_exp_* (Hz) | *J_calc_* (Hz). The ^5^*S*_1_ X-Ray geometry | *J_calc_* for equilibrium 60% ^5^*S*_1_ + 40% ^4^*C*_1_ |
| --- | --- | --- | --- |
| *J*_1,2_ | 4.8 | 3.7 | 4.0 |
| *J*_2,3_ | 9.2 | 7.7 | 9.0 |
| *J*_3,4_ | 3.5 | 0.9 | 4.5 |
| *J_4,5_* | 5.5 | 1.9 | 5.2 |

- Other compounds **13**:

For the other compounds **13**, the scalar coupling analysis strongly suggests the existence of **conformational equilibria**, since the observed values are not consistent with a single conformation (Table 3).

For the other molecules, satisfactory agreements between the experimental and expected values were obtained when the conformational equilibria between the ^2^*S*_N_,^1^*S*_3_,^1^*C*_4_ geometries were considered, depending on the particular substitution. In particular, the expected *J* values for the canonical ^2^*S*_N_,^1^*S*_3_,^1^*C*_4_,^5^*S*_1_, and ^4^*C*_1_ geometries were estimated through MSPIN and the best fit of the estimated values for a conformational equilibrium between two conformers versus the experimental *J* values was obtained. The results are summarized in Table 3. While a single major conformer was deduced for **13b**α (^4^*C*_1_), **13b**β (^2^*S*_N_) and **13f**β (^2^*S*_N_), the *J* data agreed with conformational equilibria between the ^5^*S*_1_ and ^4^*C*_1_ geometries (**13e**α) and between the ^2^*S*_N_ and ^1^*C*_4_ (**13d**b). Finally, for **13a**β, it was necessary to consider the presence of more than two conformers in equilibrium to properly interpret the observed *J* values.

Table 3. The coupling constant values (*J,* Hz) measured for the different compounds at 400 and 500 MHz in the solvents described in the Table. The deduced conformation or conformational equilibrium based on the observed *J* values are also given in the right-hand column.

| **Compound** | Experimental *J* values | | | | | | | Deduced conformation (s) |
| --- | --- | --- | --- | --- | --- | --- | --- | --- |
|  | *J*_1,2_ | *J*_2,3_ | *J*_3,4_ | *J*_4,5_ | *J*_5,6_ | *J*_5,6’_ | Other *J* values |  |
| **13a**β (500 MHz, acetone) | 3.4 | 6.1 | 6.1 | 3.3 | 5.0 | 8.3 | 4.6/6.3 (OH-6) | ^2^*S*_N_, ^1^*S*_3_, ^1^*C*_4_ |
| **13b**α (400 MHz, CDCl3) | 3.6 | 9.0 | 8.9 | 9.0 | 2.2 | 2.4 | 4.3/11.4 (OH-6) | ^4^*C*_1_. Highly predominant |
| **13b**β (400 MHz, CDCl3) | 4.4 | 8.3 | 7.5 | 3.6 | 5.6 | 5.0 | ^4^J_1,5_=0.5 (w coupling) | ^2^*S*_N_. Very major |
| **13e**α (500 MHz, CD_3_COCD_3_) | 4.8 | 9.2 | 3.5 | 5.5 | 2.8 | 5.6 |  | ^5^*S*_1_(X-Ray) + ^4^*C*_1_ (*ca.* 60:40) |
| **13f**β (400 MHz, CD_3_OD) | 5.9 | 8.5 | 8.0 | 2.8 | 6.5 | 4.9 |  | ^2^*S*_N._ Major |
| **13d**β (400 MHz, CDCl3) | 3.8 | 5.8 | 4.7 | 1.8 | 4.7 | 5.3 |  | ^1^*C*_4_, ^2^*S*_N_ |

- Compound **17**:

**7. Copies of Infra-Red Spectra**

IR of compound **2**

IR of compound **10c**

IR of compound **10f**

IR of compound **10j**

IR of compound **10m**

IR of compound **13b-α**

IR of compound **13b-β**

IR of compound **13c**

IR of compound **13f**

IR of compound **15**

IR of compound **16**

IR of compound **20**

1. [A. K. Chattopadhyay](https://onlinelibrary-wiley-com.inc.bib.cnrs.fr/authored-by/Chattopadhyay/Amit+Kumar), [V. L. Ly](https://onlinelibrary-wiley-com.inc.bib.cnrs.fr/authored-by/Ly/Vu+Linh), [S. Jakkepally](https://onlinelibrary-wiley-com.inc.bib.cnrs.fr/authored-by/Jakkepally/Shashidhar), [G. Berger](https://onlinelibrary-wiley-com.inc.bib.cnrs.fr/authored-by/Berger/Gilles), [S. Hanessian](https://onlinelibrary-wiley-com.inc.bib.cnrs.fr/authored-by/Hanessian/Stephen), *Angew. Chem. Int. Ed*. **2016**, 55(7), 2577-2581.
